# A Putative Homologue of CDC20/CDH1 in the Malaria Parasite Is Essential for Male Gamete Development

**DOI:** 10.1371/journal.ppat.1002554

**Published:** 2012-02-23

**Authors:** David S. Guttery, David J. P. Ferguson, Benoit Poulin, Zhengyao Xu, Ursula Straschil, Onny Klop, Lev Solyakov, Sara M. Sandrini, Declan Brady, Conrad A. Nieduszynski, Chris J. Janse, Anthony A. Holder, Andrew B. Tobin, Rita Tewari

**Affiliations:** 1 Centre for Genetics and Genomics, School of Biology Queens Medical Centre, University of Nottingham, Nottingham, United Kingdom; 2 Nuffield Department of Clinical Laboratory Science, University of Oxford, John Radcliffe Hospital, Oxford, United Kingdom; 3 Division of Cell and Molecular Biology, Imperial College London, London, United Kingdom; 4 Leiden Malaria Research Group, Department of Parasitology, Leiden University Medical, Leiden, The Netherlands; 5 Department of Cell Physiology and Pharmacology, College of Medicine, Biological Sciences and Psychology, University of Leicester, Leicester, United Kingdom; 6 Division of Parasitology, MRC National Institute for Medical Research, London, United Kingdom; University of Geneva, Switzerland

## Abstract

Cell-cycle progression is governed by a series of essential regulatory proteins. Two major regulators are cell-division cycle protein 20 (CDC20) and its homologue, CDC20 homologue 1 (CDH1), which activate the anaphase-promoting complex/cyclosome (APC/C) in mitosis, and facilitate degradation of mitotic APC/C substrates. The malaria parasite, *Plasmodium*, is a haploid organism which, during its life-cycle undergoes two stages of mitosis; one associated with asexual multiplication and the other with male gametogenesis. Cell-cycle regulation and DNA replication in *Plasmodium* was recently shown to be dependent on the activity of a number of protein kinases. However, the function of cell division cycle proteins that are also involved in this process, such as CDC20 and CDH1 is totally unknown. Here we examine the role of a putative CDC20/CDH1 in the rodent malaria *Plasmodium berghei* (*Pb*) using reverse genetics. Phylogenetic analysis identified a single putative *Plasmodium* CDC20/CDH1 homologue (termed CDC20 for simplicity) suggesting that *Plasmodium* APC/C has only one regulator. In our genetic approach to delete the endogenous *cdc20* gene of *P. berghei*, we demonstrate that PbCDC20 plays a vital role in male gametogenesis, but is not essential for mitosis in the asexual blood stage. Furthermore, qRT-PCR analysis in parasite lines with deletions of two kinase genes involved in male sexual development (*map2* and *cdpk4*), showed a significant increase in *cdc20* transcription in activated gametocytes. DNA replication and ultra structural analyses of *cdc20* and *map2* mutants showed similar blockage of nuclear division at the nuclear spindle/kinetochore stage. CDC20 was phosphorylated in asexual and sexual stages, but the level of modification was higher in activated gametocytes and ookinetes. Changes in global protein phosphorylation patterns in the Δ*cdc20* mutant parasites were largely different from those observed in the Δ*map2* mutant. This suggests that CDC20 and MAP2 are both likely to play independent but vital roles in male gametogenesis.

## Author Summary

Malaria parasites are single cell organisms that multiply via asexual division at different stages in the life-cycle: in the liver and red blood cells of the vertebrate host and gut of the mosquito vector. The precursor sexual stages (male and female gametocytes) form in red blood cells, then following ingestion in a blood meal the male gametocytes undergo three mitotic divisions resulting in eight male gametes in the mosquito gut. In many organisms including yeast and mice it has been shown that cell division and mitosis are tightly regulated by a set of cell division cycle proteins, namely CDC20 and CDH1. We studied the function of the single homologue of CDC20/CDH1 expressed in the rodent malaria parasite, *Plasmodium berghei*. We found that *P. berghei* CDC20 is not required for asexual multiplication but is essential for male gamete formation. Analysis of these mutant parasites revealed a defect in male gametocyte division and differentiation resulting in no male gamete formation with major defects in cytokinesis. This phenotype is similar to that of a kinase mutant (*map2*), suggesting that they play an independent but essential role in progression of the sexual stage.

## Introduction

Progression of mitosis in the cell-cycle is dependent upon a number of complex, sequential processes that are governed by a series of essential cell cycle regulatory proteins. Anaphase and mitotic exit is regulated by the conserved multi-subunit E3 ubiquitin ligase Anaphase Promoting Complex/Cyclosome (APC/C), which targets mitotic regulators such as securin and cyclin B for destruction by the 26S proteosome [1]. Two of the major regulators of APC/C activity are cell-division cycle protein 20 (CDC20) (also known as *Fizzy, p55^CDC^ or Slp1* 2–4) and its homologue, CDC20 homologue 1 (CDH1 – also known as *Cdh1p/Hct1p, Fizzy-related, Ste9, Srw1* or *Ccs52* [5]–[8]). CDC20 and CDH1 are related tryptophan-aspartic acid (WD)-40 repeat-containing adaptor proteins, which are highly conserved throughout eukaryotic evolution. They consist of approximately 40 amino acid-repeat motifs that often contain a C-terminal Trp-Asp (WD) sequence, as well as an N-terminal C-Box motif and C-terminal Ile-Arg (IR) residues, along with a KEN-box, Mad2-interacting motif (MIM) and a CRY-box [9].

CDC20 protein accumulates during S-phase, peaks in mitosis and activates the phosphorylated APC/C complex (which is phosphorylated by cyclin-dependent kinase 1 (CDK1) and other mitotic kinases [10], [11]) by physical association, which results in the activation of the metaphase-anaphase transition [12] and degradation of mitotic cyclins via ubiquitination [13]. Phosphorylation of APC/C^CDC20^ and high levels of CDK prevent CDH1 interacting with APC/C during early mitosis [14], whereas a reduction in CDK levels by the activity of APC/C^CDC20^ during telophase/G1 results in CDH1 maintaining APC/C activity and cyclin degradation in proliferating cells [6] and exit from mitosis. This shows that activity of CDC20 and CDH1 in the cell cycle is temporally controlled and ensures that exit from mitosis does not occur before sister chromatid separation has been initiated.

The activity of APC/C^CDC20^ is tightly regulated by a surveillance mechanism known as the spindle assembly checkpoint (SAC) [15]. The SAC is a pathway that prevents the unregulated separation of sister chromatids [16] and consists of a number of regulatory proteins including mitotic-arrest deficient (MAD) 1, MAD2, MAD3, budding uninhibited by benzimidazoles (BUB) 1 and BUBR1. The SAC negatively regulates the activity of APC/C^CDC20^ by preventing ubiquitination of securin and cyclin B and subsequently prolongs prometaphase until all chromosomes have been correctly oriented [15]. This process occurs at the kinetochores, where MAD2 and BUBR1 interact with APC/C^CDC20^ to form a mitotic checkpoint complex (MCC) [17], which inhibits its activity. Once the chromatids are correctly oriented, APC/C^CDC20^ becomes active as it is released from the MCC and initiates anaphase, degrading securin and cyclin B and resulting in reduced CDK activity. This reduction in kinase activity promotes the formation of APC/C^CDH1^ and results in exit from mitosis via degradation of APC/C^CDC20^, maintaining cyclin degradation in G1 prior to a new round of DNA replication [18], [19].

Regulation of the cell-cycle and DNA replication in the unicellular apicocomplexan malaria parasite, *Plasmodium*, is known to be highly complex and dependent on the activity of a number of protein kinases [20]. *Plasmodium* is a haploid organism lacking sex chromosomes but with a complex life-cycle involving both asexual and sexual processes. Asexual multiplication occurs at three particular stages of the parasites life-cycle: blood stage schizogony, sporogony in the mosquito and pre-erythrocytic schizogony in liver hepatocytes [21]. As with some, but not all apicomplexan parasites multiplication involves repeated nuclear divisions before daughter formation by a process termed schizogony. During these stages genome duplication and segregation is accomplished using an intra-nuclear spindle while retaining an intact nuclear membrane without the formation of the typical morphological features of mitosis [22], [23]. In contrast, DNA replication during *Plasmodium* sexual stages within male gametocytes occurs in the mosquito vector and involves three rounds of genomic replication resulting in eight microgamete nuclei and ultimately eight microgametes [24]–[27].

Upon ingestion of a blood meal by the female *Anopheles* mosquito, exposure of the male gametocyte to a slight drop in temperature, a rise in intracellular Ca^2+^ concentration and the mosquito-derived metabolic intermediate xanthurenic acid [28]–[30] result in rapid DNA replication (within 12 min) and mitosis giving rise to eight gametes, which egress out of the cell in a process termed exflagellation. This process is known to be dependent upon two protein kinases – calcium-dependent protein kinase 4 (CDPK4) and mitogen-activated protein kinase 2 (MAP2) [20], [28], [31]–[33]. Activation of CDPK4 results in genome replication, mitosis and axoneme assembly [28] and in a subsequent step; MAP2 is activated and results in axoneme motility and cytokinesis [32]. However, the cell division cycle proteins that interact with these kinases are unknown. As described earlier, in human and yeast cells CDC20 and CDH1 are known to play a major part in cell cycle regulation [9] particularly during early mitosis, and interact with regulatory kinases and phosphatases [3], [7], [34].

To examine the function of a single homologue of CDC20/CDH1 (termed CDC20 for simplicity) in the complex life-cycle of *Plasmodium* we used a rodent malaria model, *P. berghei (Pb)* in laboratory mice, which is very amenable to analysis by reverse genetics and where the entire life cycle, including within the mosquito vector, can be analysed. The results presented here suggest that CDC20 has an essential role in *Plasmodium* male gamete formation, possibly through interacting with the kinase regulator MAP2, but has no essential involvement in asexual multiplication.

## Results

### *Plasmodium* has a single homologue for CDC20/CDH1

**Figure 1:**
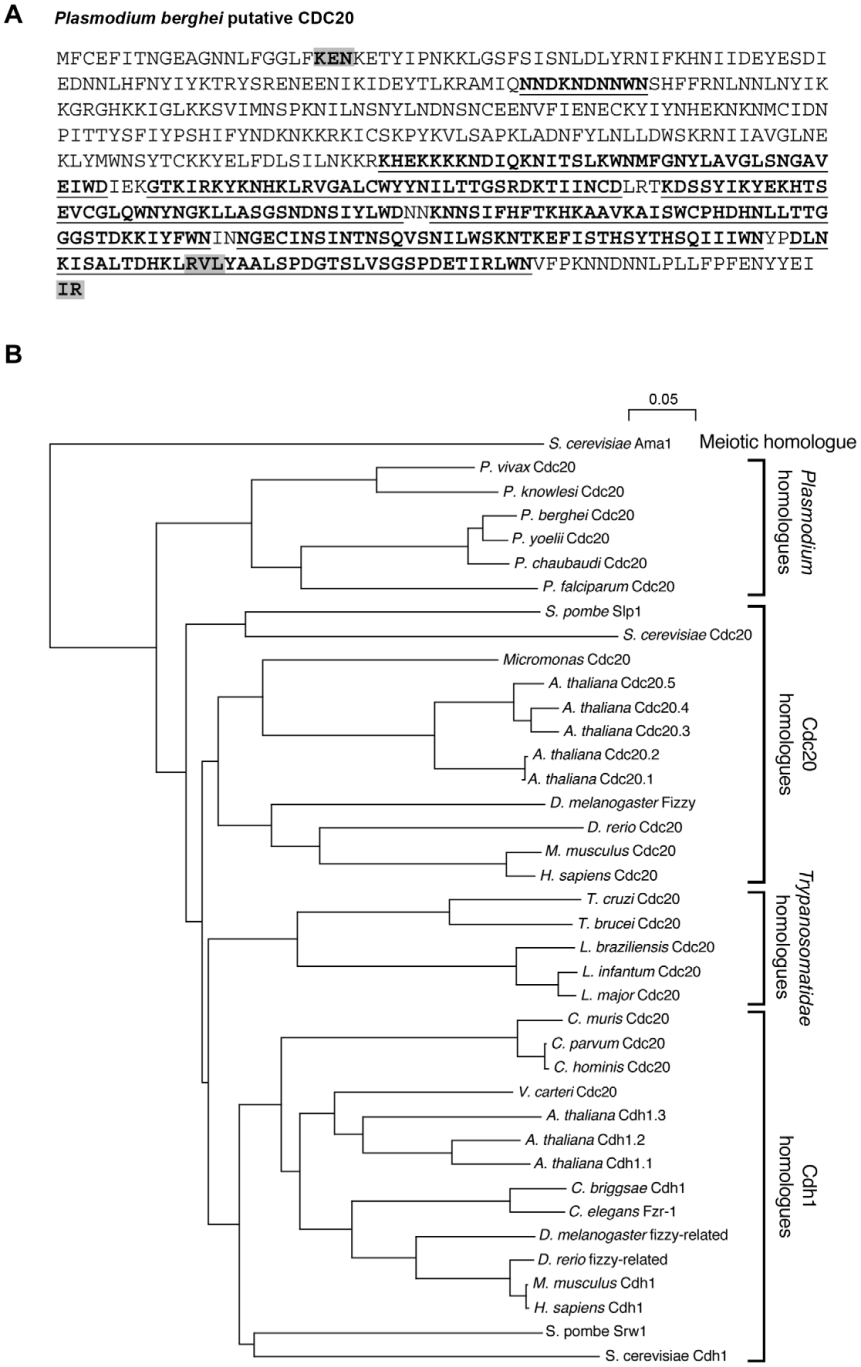
Phylogenetic analysis of *Plasmodium* CDC20. A. Amino-acid sequence of a *Plasmodium berghei* putative CDC20. WD repeats are shown in bold and underlined. Other motifs (highlighted) are the KEN box, RVL cyclin binding motif and IR motif. B. WD domains from various eukaryotic Cdc20 and Cdh1 homologues were aligned and used to draw a phylogenetic tree. The *S. cerevisiae* meiotic APC/C regulator (Ama1) was used as an out group. Four clusters are apparent. Two correspond to previously described Cdc20 and Cdh1 protein families. A third includes homologues from *Trypanosomatidae* species. The fourth cluster includes all the identified CDC20/CDH1 homologues from *Plasmodium* species.

Sequence analyses of *P. berghei* identified a *cdc20* gene (PBANKA_051060) comprised of one exon. The protein contains a classical KEN-box, RVL-cyclin binding motif, IR motif and seven WD-40 repeat motifs as found in CDC20 and CDH1 of other organisms (Figure 1A), but does not contain a C-box, D-box or a Mad2-interacting motif. We were only able to identify a single CDC20/CDH1 homologue coded in the genomes of *Plasmodium* species, which has also been suggested for *Trypanosomatidae* [35]. To assess the evolutionary relationships between these CDC20 homologues we aligned (using ClustalW – Figure S1) the WD domains and used the alignment to draw a phylogenetic tree using the meiotic APC/C activator from yeast as an out group (Figure 1B). In the resulting tree we see four clusters. Two clusters, as expected, represent the CDC20 and CDH1 homologues from a range of eukaryotic species. Another cluster contains the CDC20/CDH1 homologues from *Trypanosomatidae* species. The final cluster includes all the CDC20/CDH1 homologues from *Plasmodium* species. These results suggest that *Plasmodium* species contain only a single CDC20/CDH1 homologue and that the *Plasmodium* APC/C has only one regulator.

### CDC20-GFP shows nuclear expression through-out the life-cycle, with highest expression in activated male gametocytes

**Figure 2:**
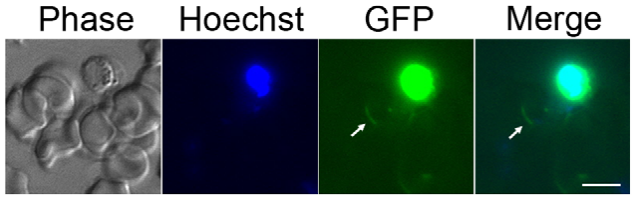
CDC20-GFP protein expression in activated male gametocytes. High CDC20-GFP intensity was observed in activated male gametocytes and co-localised with Hoechst nuclear staining in both the microgametocyte body and exflagellating microgametes (arrows). Bar = 5 µm.

Little information is available regarding CDC20 expression and localisation in the malaria parasite in both vertebrate and mosquito hosts. Therefore, we generated a C-terminal green fluorescent protein (GFP) fusion protein from endogenous *cdc20* using a single crossover recombination strategy (Figure S2A–D). Correct targeting was confirmed using integration PCR and Southern blot (Figure S2B, C). Expression of CDC20-GFP in transgenic parasites was confirmed by Western blotting using an anti-GFP polyclonal antibody (Figure S2D). A protein band of ∼92 kDa was present for all analysed CDC20-GFP samples, which corresponds to the predicted mass of the CDC20-GFP fusion protein (92.4 kDa). The line expressing the unfused GFP [36] produced a band at 29 kDa and was used as a control (Figure S2D). Expression of the CDC20-GFP fusion protein resulted in no visible abnormalities. Low intensity CDC20-GFP expression was detected during all stages of the life-cycle (data not shown) apart from activated male gametocytes, which had the highest intensity of GFP expression that co-localized with Hoechst nuclear staining (Figure 2). We also generated a parasite line whereby CDC20-GFP was expressed episomally (using the same plasmid utilised to target the endogenous locus) under the control of the *cdc20* promoter. This line showed high GFP fluorescence intensity at all stages, which co-localised with Hoechst nuclear staining in asexual, gametocyte and oocyst stages and an additional cytoplasmic localisation in ookinetes (Figure S3).

### CDC20 is critical to male gamete formation and exflagellation

To discover the function of CDC20 in the *Plasmodium* life-cycle, we used a double crossover homologous recombination strategy to knockout the gene. This was achieved by replacing the endogenous gene with a pyrimethamine resistant allele of the dihydrofolate reductase-thymidine synthetase (*dhfr*/*ts*) gene from *Toxoplasma gondii* (Figure S2E). Successful integration of the gene was confirmed by a diagnostic PCR across the junction of the expected integration site, as well as by Southern blot, pulsed-field gel electrophoresis (PFGE) and quantitative reverse transcription PCR (qRT-PCR) to indicate an absence of transcription (Figure S2F–I).

**Figure 3:**
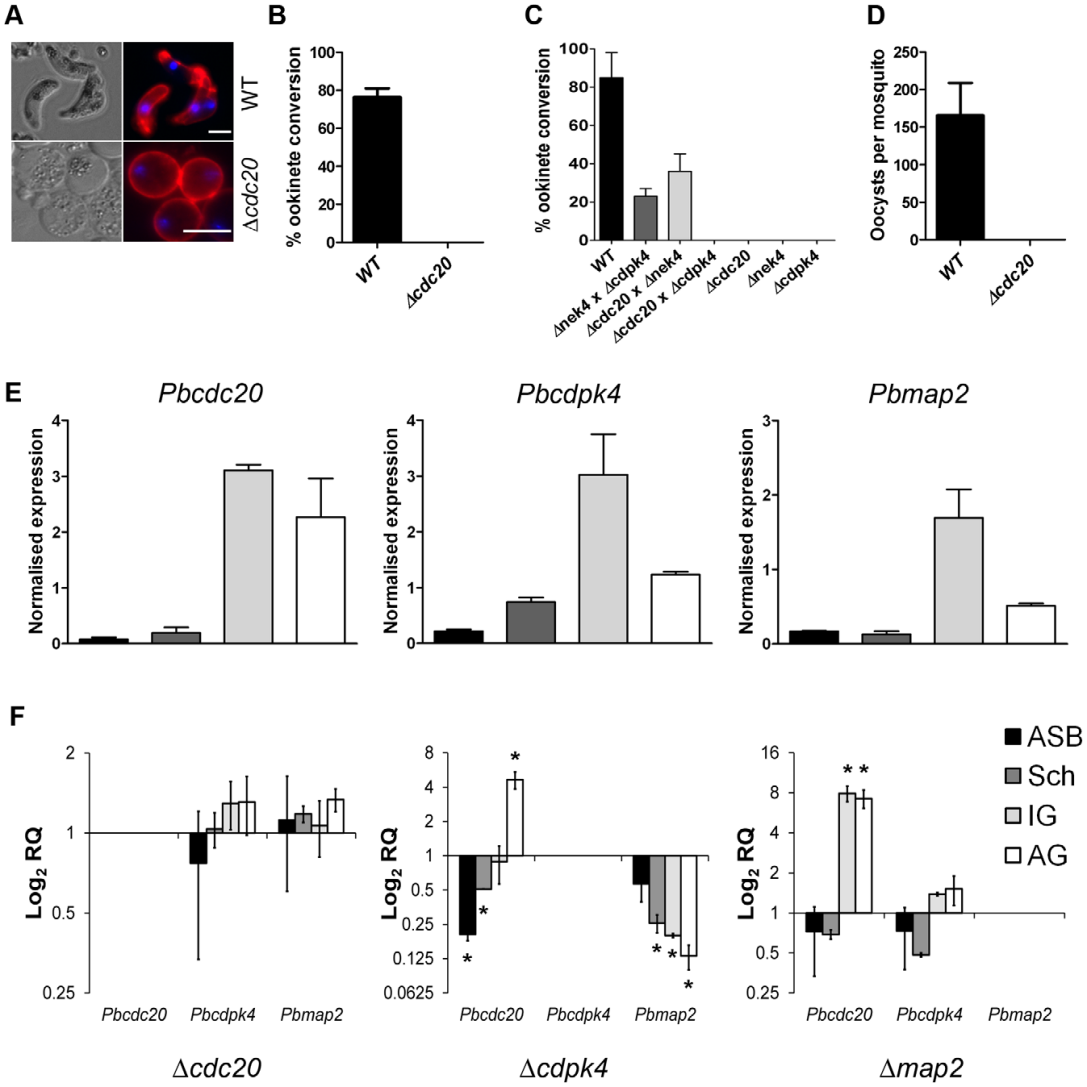
Phenotypic analysis of Δ*cdc20*. A. Immunofluorescence images of *Plasmodium* cultures after 24 hr *in vitro* immunostained for the female gamete/zygote/ookinete marker P28 (red) and counterstained with the nuclear marker Hoechst (blue). Development of elongated ookinetes was completely ablated in Δ*cdc20* lines, which produced only round female gametes. Bar = 5 µm. B. Bar graph illustrating ookinete conversion in wild-type and Δ*cdc20* parasites. The conversion rate is the percentage of P28-positive parasites that had successfully differentiated into elongated ‘banana-shaped’ ookinetes (error bar = arithmetic mean ±SD; *n* = 3). C. Ookinete conversion after crossing Δ*cdc20* parasites with a female-defective *nek4* mutant (Δ*nek4*) and a male-defective *cdpk4* mutant (Δ*cdpk4*). Wild-type parasites were used as a control. Bar graph represents the percentage of round P28-positive parasites that had converted into elongated ookinetes (arithmetic mean ±SD; *n* = 3). D. Bar graph showing average numbers of oocysts per gut (error bar indicates ±SEM; *n = *60 of wild-type or Δ*cdc20* infected mosquitoes from three independent experiments). Overall infection prevalence was 80% for wild-type and 0% for Δ*cdc20*. E. Wild-type mRNA expression of *cdc20*, *cdpk4* and *map2* relative to *hsp70* and *arginyl-tRNA synthetase* as endogenous controls (ΔΔCt method). Error bars represent ±SEM, *n = *3 from three independent experiments. The key to the shading of bars is indicated in F. F. Relative expression of *cdc20*, *cdpk4* and *map2* in Δ*cdc20*, Δ*cdpk4* and Δ*map2* parasites compared to wild-type parasites (Pfaffl method). Error bars represent ±SEM, *n = *3 from three independent experiments. ASB  =  Asexual blood; Sch  =  Schizont; IG  =  Inactivated gametocytes; AG  =  Activated gametocytes; RQ  =  relative quantification.

Analysis of two *cdc20* deletion mutant clones, N10 cl7 and N10 cl9 (henceforward called Δ*cdc20*), showed no developmental abnormalities during asexual proliferation or gametocyte formation, as assessed on blood smears (data not shown). However, *in vitro* cultures analysed for differentiation into ookinete stages [20], [37] showed complete ablation of ookinete development (Figure 3A, B). To ascertain whether the block in ookinete formation was a defect along the male or female line, we performed genetic crosses as previously described [37], [38]. Crossing of Δ*cdc20* with a *cdpk4* deletion mutant (a previously characterised male mutant [28], henceforward called Δ*cdpk4*) showed no rescue of the phenotype. Conversely, crossing with a *nek4* deletion mutant (a previously characterised female mutant [38], henceforward called Δ*nek4*) resulted in 36% ookinete formation (Figure 3C). These data prove that Δ*cdc20* parasites are defective along the male line. As a result of this observation, we analysed exflagellation of the activated male gametocytes [26], which was completely blocked in Δ*cdc20* parasites. To substantiate the *in vitro* findings, we fed mosquitoes on mice infected with either wild-type or Δ*cdc20* parasites and analysed oocyst development. Wild type parasites developed normally and oocysts were detected in the mosquito gut, whereas no oocysts were found in the guts of mosquitoes fed on Δ*cdc20* parasites and analysed 14 or 21 days after feeding (Figure 3D). This result confirms that CDC20 is vital to male gamete development and that fertilization/zygote formation/ookinete development is completely blocked in the Δ*cdc20* parasites, preventing oocyst formation.

### Expression of cdc20 is up-regulated in Δ*cdpk4 and* Δ*map2* mutants

Exflagellation of the activated microgametocyte proceeds via a number of sequential steps prior to the formation of male gametes [24], [25]. These steps are dependent upon two protein kinases; calcium-dependent protein kinase 4 (CDPK4), which is involved in cell-cycle progression to S phase and mitogen-activated kinase 2 (MAP2), which is essential for replication and mitosis to be completed before cytokinesis commences [20], [28], [31]–[33]. Both of these kinases have previously been shown to be essential for male gamete development and the exflagellation process [28], [32]. As the *cdc20* deletion mutant line shows a similar phenotype, we decided to analyse mRNA expression of *cdc20*, *map2* (PBANKA_093370) and *cdpk4* (PBANKA_061520) in our Δ*cdc20* line as well as the previously characterised Δ*map2* and Δ*cdpk4* mutant lines.

Transcription of these three genes in total asexual blood, schizont and gametocyte stages of wild type parasites showed a similar profile, with highest mRNA levels found in gametocytes (Figure 3E). When compared to wild-type, expression of both *map2* and *cdpk4* was not significantly altered at any stage in the Δ*cdc20* mutant; however, striking differences were found in *cdc20* mRNA levels in both the Δ*cdpk4* and Δ*map2* mutants. *cdc20* was found to be significantly down-regulated in Δ*cdpk4* asexual blood and schizont stages (*p = *0.037 and 0.009 respectively). In contrast, expression in activated Δ*cdpk4* activated gametocytes was significantly up-regulated (*p = *0.001), but was not altered in non-activated blood stage gametocytes. The greatest change in *cdc20* expression was observed in both non-activated and activated gametocyte stages of the Δ*map2* parasites, where expression was significantly up-regulated (*p = *<0.001 and 0.001 respectively). Expression of *map2* in the Δ*cdpk4* line was shown to be significantly down-regulated in schizont and non-activated and activated gametocyte stages (*p = *<0.01 for all), whereas no significant alteration in *cdpk4* was observed at any stage of the Δ*map2* parasites analysed (Figure 3F).

### CDC20 is not essential for genome replication in activated microgametocytes, but regulates cytokinesis and subsequent exflagellation

**Figure 4:**
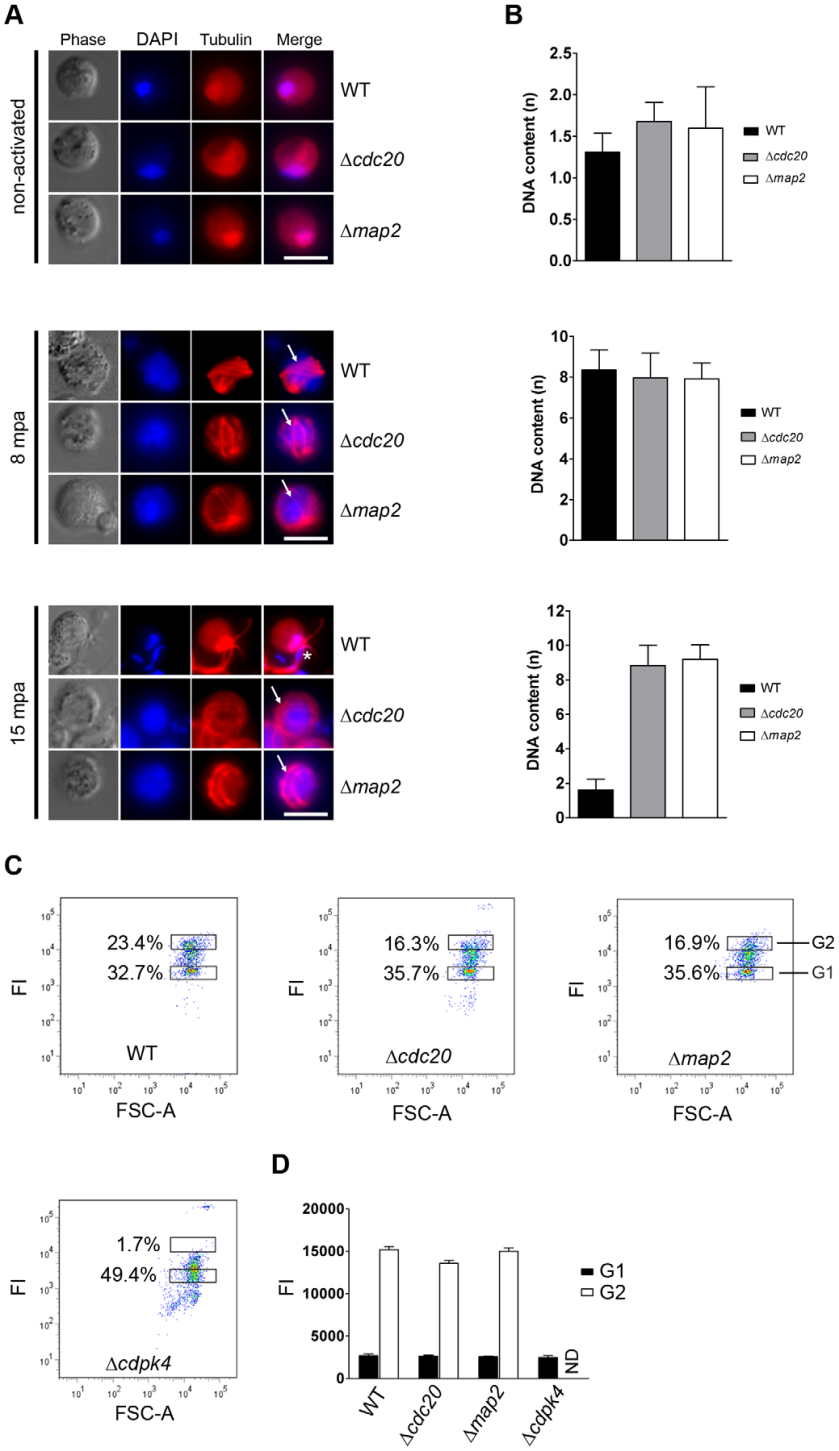
Analysis of genome replication in activated male gametocytes by direct (immuno) fluorescence microscopy and FACS analysis. A. Direct immunolabelling of α-tubulin (red) and DNA (Hoechst – blue) in activated gametocytes fixed at different time points post activation (pa). Representative cells from one of three experiments are shown. The 8 min time point shows characteristic axonemic circling of the nucleus (arrow) whereby each of the 8 axonemes lie completely within the cytoplasm, coiled around the nucleus. The 15 min time point illustrates an exflagellating wild-type microgametocyte with condensed DNA entering the flagella (indicated by *). Δ*cdc20* and Δ*map2* parasites are arrested at the exflagellation stage. Bar = 5 µm. mpa  =  minutes post-activation. B. Fluorometric anaylsis of DNA content (n) after DAPI nuclear staining. Microgametocytes were at 0 mpa (non-activated), 8 mpa or 15 mpa. The mean DNA content (and standard deviation) of 10 nuclei per sample are shown. Values are expressed relative to the average fluorescence intensity of 10 haploid ring-stage parasites from the same slide [69]. All values were corrected for background fluorescence. C. Determination of DNA content of purified, activated male gametocytes at 8 mpa by FACS analyses of Hoechst-stained parasites [63]. Dot plots show the mean percentage of gametocytes in gate G1 (inactivated and activated female gametocytes) and G2 (activated gametocytes with an 8n DNA content. Wild-type, Δ*cdc20* and Δ*map2* parasites show a high percentage of activated male gametocytes with an 8n DNA content (see D). The previously characterised Δ*cdpk4* parasites [28] were used as a control as they do not undergo DNA replication upon activation. FI  =  Fluorescence Intensity. D. Mean Hoechst fluorescence intensity (DNA content) (±SD) of gametocytes in gates G1 and G2 in three independent experiments. The DNA content of Δ*cdc20* and Δ*map2* male gametocytes (gate G2) at 8 mpa is comparable to that of wild-type gametocytes whereas activated, DNA replicating males are absent in Δ*cdpk4* parasites. FI  =  Fluorescence Intensity; ND  =  not determined.

Due to the ablation of exflagellation in the Δ*cdc20* line and the significant alteration in *cdc20* expression in the Δ*map2* line, we analysed axoneme formation and DNA replication in both mutants by direct immunofluorescence and fluorometric estimation of DNA content respectively. Staining of α-tubulin in both Δ*cdc20* and Δ*map2* lines revealed normal formation of axonemes and their characteristic circling of the nucleus by the axonemes in concentric rings 8 min post activation (mpa) (Figure 4A). However, differentiation and shortening of the spindle microtubules did not occur in either mutant 15 mpa. Furthermore, nuclear DNA in the enlarged nucleus of activated microgametocytes remained uncondensed in both mutants at 15 mpa; whereas wild-type microgametocytes had started to undergo exflagellation and nuclear divison resulting in the release of normal microgametes containing haploid nuclei with condensed DNA. These observations suggest that development of mutant microgametocytes after activation is blocked at a very late stage, possibly after the third round of DNA replication.

To test whether mutant microgametocyte development was blocked after completing the three rounds of DNA replication, we analysed DNA replication by determination of the DNA content of activated microgametocytes by fluorescence microscopy and by FACS after staining with the DNA-specific dyes 4,6-diamidino-2-phenylindole (DAPI) and Hoechst 33258, respectively. The DNA content of activated microgametocytes at 8 mpa, as determined by fluorescence microscopy, was similar in wild-type and mutant parasites, with nuclei of mutant parasites also increasing their DNA content to the octoploid level at 8 mpa (Figure 4A, B; upper and middle panels). At 15 mpa the activated Δ*cdc20* and Δ*map2* microgametocytes still contained a single enlarged nucleus with octoploid DNA content, but in contrast, in wild type microgametocytes nuclear division and gamete formation resulted in the formation of gametes with haploid DNA content (Figure 4A, B; lower panels). Genome replication in activated microgametocytes was confirmed using FACS analysis of purified gametocytes that were stained with Hoechst 33258. At 8 mpa both Δ*cdc20* and Δ*map2* microgametocytes showed strongly increased DNA content similar to that of wild-type parasites (Figure 4C, D). Purified gametocytes of the previously characterised Δ*cdpk4* parasite line [28] were used as a control and did not undergo DNA replication. Together these results suggest that CDC20 acts downstream of CDPK4 and has an essential role in axoneme motility, DNA condensation and cytokinesis, similar to MAP2 [32], but does not play a role in activation of genome replication.

### *cdc20* mutants show defects in nuclear pole and kinetochore progression

**Figure 5:**
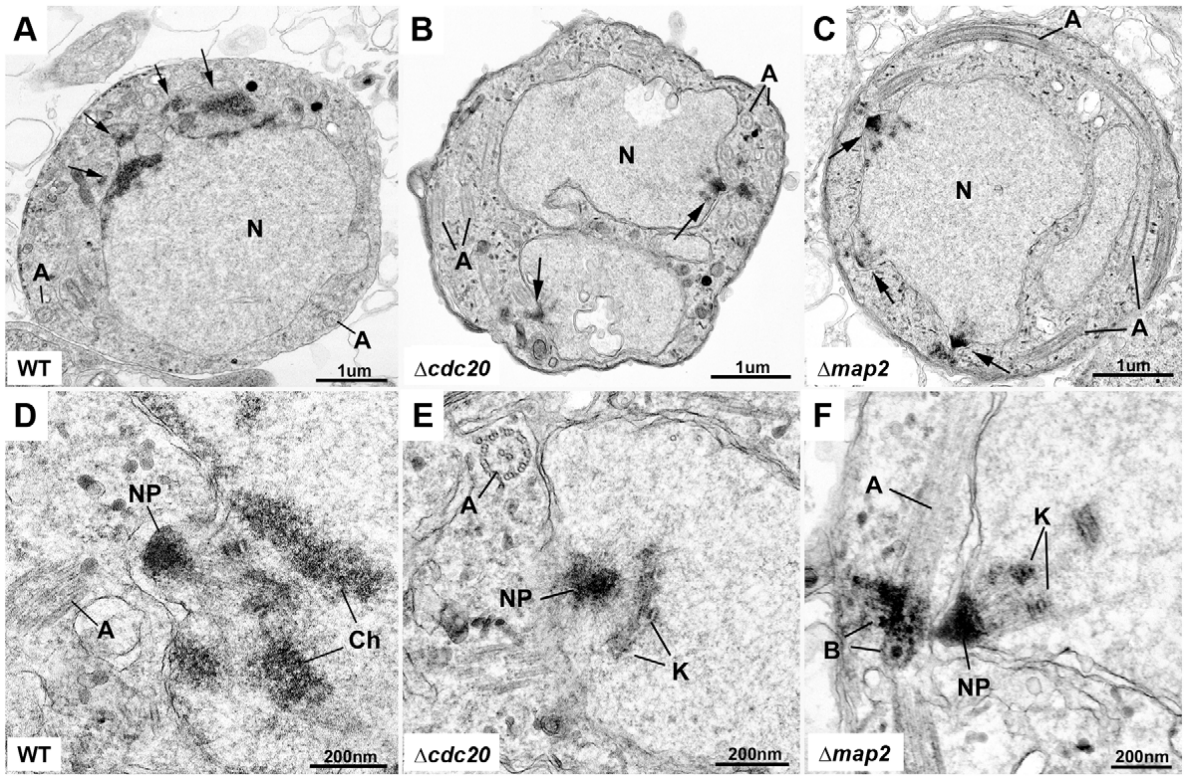
Ultrastructural analysis of Δ*cdc20* and Δ*map2* parasite lines. Electron micrographs of wild-type (A, D), Δ*cdc20* (B, E) and Δ*map2* (C, F) gametocytes 15 min after activation. A. Late stage wild-type microgametocyte exhibiting areas of electron dense chromatin (arrows) budding from the nucleus (N). A  =  axonemes. B and C. Mutant microgametocytes at an early stage in microgametocyte development showing multiple nuclear poles (arrows) with radiating microtubules. N  =  nucleus; A  =  axoneme. D. Detail from A. showing the nuclear pole (NP) and areas of electron dense chromatin (Ch). A  =  axoneme. E and F. detail from the two mutants (B, C) showing the nuclear pole (NP) with radiating microtubules and attached kinetochores (K) but an absence of condensed chromatin. A  =  axoneme; B  =  basal body.

Due to the similar morphology and dynamics of DNA replication of *cdc20* and *map2* mutants as analysed by direct immunofluorescence and DNA content analysis, respectively, we next examined whether deletion of the endogenous *cdc20* locus resulted in structural defects that were similar to those associated with the *map2* mutant line by electron microscopy. Ultrastructure analyses were performed on wild-type, Δ*cdc20* and Δ*map2* gametocytes at 15 and 30 mins after activation. The appearance of the cytoplasm was similar for all three lines with the formation of a number of axonemes (Figure 5A, B, C). The microgametocyte nucleus also appeared similar in all three strains with homogeneous electron lucent nucleoplasm and the formation of nuclear poles with radiating microtubules and attached kinetochores (Figure 5B, C, E, and F). However, only in the wild-type was it possible to observe nuclear poles with condensed chromatin consistent with later stages in microgamete nucleus formation (Figure 5A, D), whereas the mutants showed defects in chromosome condensation.

To identify any quantitative differences, 100 parasites of each line and time point were examined with nuclear appearance divided into four categories. When the number of microgametocytes displaying the various nuclear appearances was counted, while the mutants appeared similar, significant differences were observed between the mutants and the wild type (Table 1). It was observed that wild-type parasites exhibited all stages of microgamete nuclear development with reduced numbers of the early stages with tubules and kinetochores (21% compared to 10%) and increased numbers of the later stages with condensed chromatin (42% compared to 29%) at 30 mins compared to 15 mins post-activation (Table 1). In both mutants approximately half the nuclei exhibited early stage microtubules and attached kinetochores (49–60%) and parasites with dense nuclear pole or chromatin condensation (0–3%) were rarely observed irrespective of the time point (Table 1). While no structural abnormality was observed, the quantitative differences are consistent with the two mutants being “frozen” at the nuclear spindle kinetochore formation stage. Furthermore, chromosome condensation was not observed in either *cdc20* or *map2* mutants as compared to wild type parasites.

**Table 1:** Nuclear features of wild-type and mutant parasites based on stage of microgametocyte development.

Strain	Time (min)	No features^1^	Early^2^	Mid^3^	Late^4^
Wild type	15	35	21	15	29
Δ*cdc20*	15	46	51	2	1
Δ*map2*	15	40	60	0	0
Wild type	30	33	10	15	42
Δ*cdc20*	30	40	58	2	0
Δ*map2*	30	48	49	3	0

Quantitation of the nuclear features observed by electron microscopy was carried out at the 15 and 30 minute time points. This was based on the examination of 100 microgametocytes identified by axoneme formation at each time point. The features identified were nuclei with.

1 no specific features in the plain of section,

2 early stage exhibiting nuclear poles with spindle microtubules and kinetochores,

3 mid stage with nuclear pole but no attached kinetochores, and.

4 late stage with the nucleus exhibiting areas of condensed chromatin. Microgametogenesis is dynamic process and nuclear changes will relate to the length of time spent in each phase allowing the identification of any differences between parasite lines.

### Phosphorylation of CDC20 during gametogenesis

**Figure 6:**
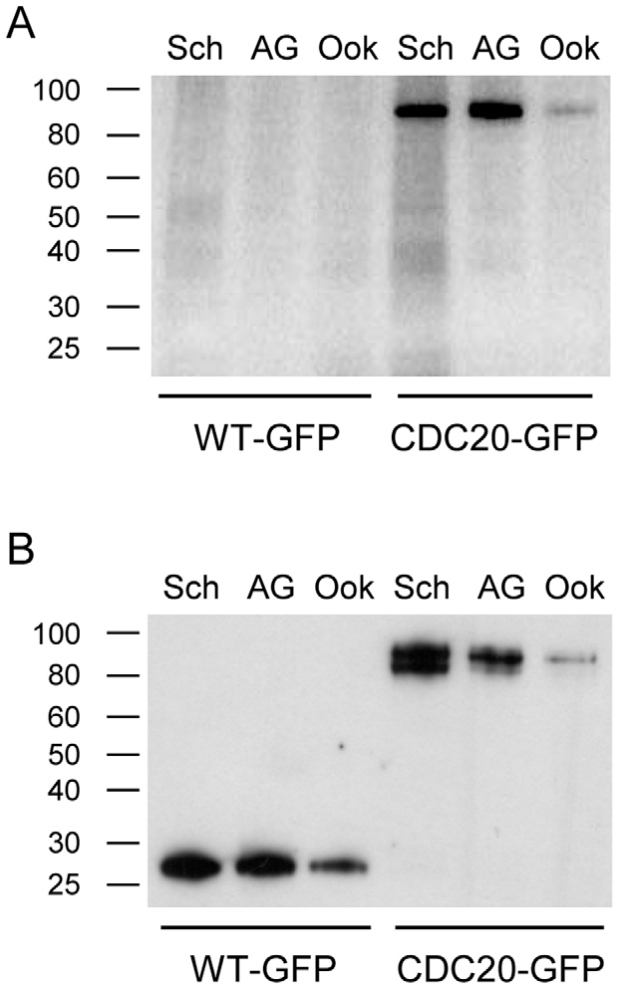
Phosphorylation of CDC20 in schizonts, activated gametocytes and ookinetes. Schizonts (Sch), activated gametocytes (AG) and ookinetes (Ook) purified using the corresponding Nycodenz protocols were metabolically labelled with ^32^P-orthophosphate for 30 min, lysed, and GFP-tagged CDC20 was immunoprecipitated using GFP-TRAP beads. A. Phosphorylation of CDC20-GFP in schizonts, activated gametocytes and ookinetes as assessed by autoradiography. B. Protein expression levels by Western Blot using a polyclonal anti-GFP antibody.

Reversible phosphorylation is an important regulatory mechanism in mitotic progression. In human and yeast cells, phosphorylation of CDC20 is known to be an essential step during anaphase and early mitosis [11], [39]. As exflagellation of Δ*cdc20* parasites is completely ablated, but DNA replication and axoneme motility in activated microgametocytes was indistinguishable from wild-type parasites, we hypothesised that phosphorylation of CDC20 could be a vital regulator of *Plasmodium* gametogenesis. Analysis of CDC20 phosphorylation was performed before, during and after completion of microgametogenesis (i.e. in schizont, activated gametocyte and ookinete stages respectively) in CDC20-GFP parasites metabolically labelled with ^32^P-orthophosphate [40] and immunoprecipitated by GFP-trap. ^32^P-orthophosphate labelling in whole cell lysates of schizonts, activated gametocytes and ookinetes showed similar profiles and confirmed efficient uptake of ^32^P-orthophosphate in all stages. Autoradiography showed that CDC20 is phosphorylated at all three stages (Figure 6A) but phosphorylation levels were higher in activated gametocytes and ookinetes compared to schizonts (1.70 and 2.48 times higher respectively) (Figure 6A, B). The GFP-tagged CDC20 protein appeared as a doublet by Western Blot in schizonts (Figure 6B), whereas only a single band was detected on the corresponding autoradiograph (Figure 6A), suggesting that the upper band on the Western Blot may represent a phosphorylated form of CDC20-GFP and the lower band a non-phosphorylated form of the protein. Interestingly, in activated gametocytes and ookinetes, only the upper GFP-immunoreactive band is present, which may reflect a higher degree of phosphorylation of CDC20-GFP in sexual stages compared to schizonts.

### Deletion of *cdc20* and *map2* affects specific protein phosphorylation in activated gametocytes

**Figure 7:**
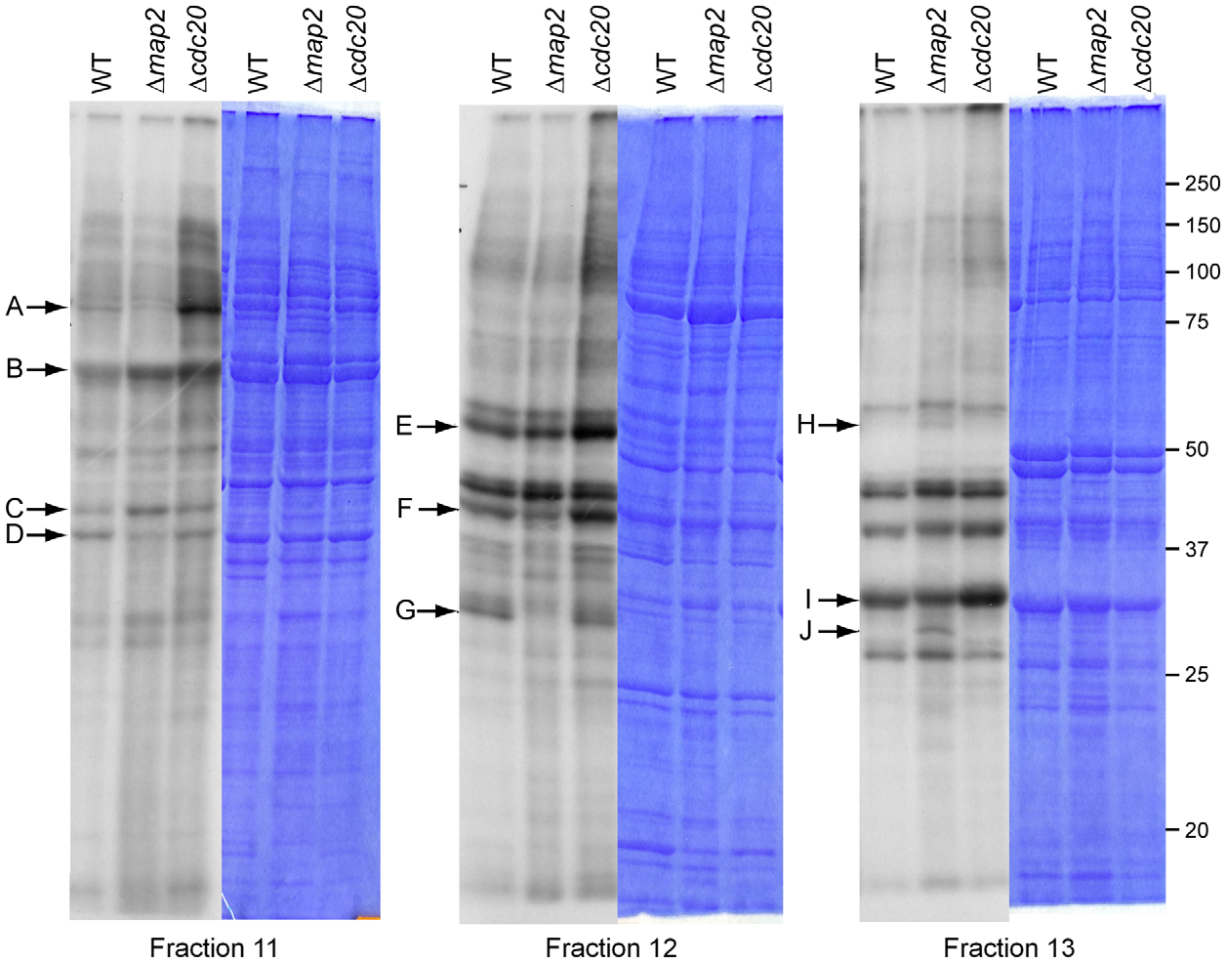
Global phosphorylation in Δ*cdc20* and Δ*map2* lines. Gametocytes from wild type, Δ*map2* and Δ*cdc20* parasites were purified on 48% Nycodenz and activated for 30 mins in ookinete medium before addition of ^32^P-orthophosphate for 30 mins. After washing, labelled activated gametocytes were lysed with NP40 and fractionated using anion exchange chromatography on an AKTA system. Individual fractions were then further resolved by SDS-PAGE and labelled bands detected by autoradiography. The Coomassie blue stained gel shows that protein loading was similar between lanes. Several differences in the ^32^P signal (indicated by arrows) are observed between the three different parasites. Bands C, D, F, G H and J indicate altered phosphorylation status only in the Δ*map2* mutant. Bands A, E and I indicate changes only in the Δ*cdc20* mutant. Only one band (band B) showed a similar change in both the Δ*map2* mutant and Δ*cdc20* mutant.

In order to examine whether or not CDC20 has a role in pathways of protein phosphorylation similar to those of the kinase MAP2, we compared the global phosphorylation profile of wild type activated gametocytes with that of Δ*cdc20* and Δ*map2* lines using metabolic labelling with ^32^P-orthophosphate [40]. This approach employs metabolic labelling of parasites followed by fractionation by ion exchange chromatography. The experiment was performed in triplicate and in each experiment 20 fractions were collected, resolved by SDS-PAGE and an autoradiograph obtained for seven of them to reveal the phosphorylation profile. Shown in Figure 7 are three fractions from the ion exchange fractionation where differences in the phosphorylation profile between the wild type and mutant parasite strains were observed. Importantly, the Coomassie blue stain of the SDS-PAGE gels demonstrated that the overall protein expression profiles of the wild type and mutant parasite lines were very similar (Figure 7). Despite this similarity, there were clear differences in the phosphorylation profile between the parasite lines. The phosphorylated band labelled A in Figure 7 was significantly decreased in the Δ*map*2 mutant, whereas the Δ*cdc20* mutant showed increased phosphorylation. Bands C, D, F, G, H and J showed altered phosphorylation status only in the Δ*map2* mutant, whereas bands A, E and I were changed only in the Δ*cdc20* mutant. Only one band (band B) showed a similar change in both the Δ*map2* and Δ*cdc20* mutants. This analysis indicated that although the phosphorylation profile of the parasite was affected by the deletion of *map2* and *cdc20*, the proteins that showed a change in phosphorylation status in the two mutant lines were (with the exception of one protein) different. It seems unlikely therefore that MAP2 and CDC20 regulate the same network of phospho-proteins. We are currently investigating this result further using mass spectrometry-based phosphoproteomic approaches.

## Discussion

Mechanisms to control cell division and the cell cycle are essential parts of the cell regulation machinery. These processes are not well understood in unicellular protozoa such as the malaria parasite *Plasmodium*. *Plasmodium* undergoes two distinct mitotic processes; one involving repeated DNA duplication, in which karyokinesis occurs after each replication and is associated with asexual proliferation and the other involving endoreduplication, with three rounds of replication prior to the simultaneous formation of eight microgamete nuclei during microgametogenesis. Here, we describe a CDC20/CDH1 orthologue in *Plasmodium* as an important regulator of mitosis during male gametogenesis, but interestingly it has no effect on the mitotic process undergone during schizogony.

Our bioinformatic studies suggest that in *Plasmodium* there is only one gene representing CDC20 and its homologue CDH1, and that the protein is a true structural homologue of CDC20/CDH1, even though we could not complement CDC20 function in yeast (data not shown). Although we cannot exclude the possibility that we failed to detect a second highly spliced *Plasmodium cdc20/cdh1* homologue, the phylogenetic clustering of all the *Plasmodium* CDC20 homologues gives confidence that there is only a single CDC20 orthologue in *Plasmodium* species. This suggests that *Plasmodium* diverged from other eukaryotes prior to the duplication event that presumably gave rise to CDC20 and CDH1 genes. It is interesting to note that the *Plasmodium* cluster is distinct from the *Trypanosomatidae* cluster where there is also a single corresponding gene in each genome. Furthermore, this orthologue has a classical KEN box-like domain at the N-terminus and an RVL domain and IR motif at the C-terminus, all of which are required for cyclin degradation and binding to the APC/C core [7]. The presence of these domains suggests that CDC20 in *Plasmodium* could influence the cell cycle in a similar manner to other systems, such as yeast, mammals and plants [9], [41]. The lack of a D-box and presence of a KEN-box are consistent with the structure of CDC20 in humans, with the presence of a KEN-box suggesting that *Plasmodium* CDC20 is a prime target for ubiquitination, as suggested in a recent study [42]. Alternatively, as *Plasmodium* CDC20 is the only orthologue of both CDC20 and CDH1 present in other systems, it is plausible that ubiquitination of CDC20 in *Plasmodium* is self-regulating, as CDC20 is known to be degraded by APC/C^CDH1^ via its KEN-box [43] and could therefore act as a “negative feedback” mechanism as seen in human cells [44], [45]. The seven conserved WD repeats in the *Plasmodium* CDC20 protein also suggests that it does bind an as yet unknown multi-protein complex. *Plasmodium* CDC20 shows some differences from the *ccs52* homologue reported in plants, such as *Medicago sativa* [8], since it lacks a MAD-binding box and also the D-box that appears to be specific for CDH1 and is not conserved in CDC20 and FZY proteins. It has been reported recently that in *Arabidopsis thaliana* there are five isoforms of CDC20, and two of them are functional [41]. We did not observe any such expansion of genes for this protein in *Plasmodium*.

Our CDC20-GFP expression studies showed that CDC20 is highly expressed in activated male gametocytes (with gametocytes showing highest expression at the mRNA level, in agreement with previous transcriptomic studies [46]) but it is also present throughout the life-cycle and located mainly in the nuclear compartment, with some cytoplasmic localisation, consistent with expression in other systems [47], [48]. However, although previous studies have shown *cdc20* transcripts and protein to be highly expressed in sporozoites of *P. falciparum* [46], [49], we did not observe high protein expression levels of CDC20-GFP in sporozoites.

Functional studies using a gene deletion strategy showed that CDC20 controls male gamete development and deletion mutants are impaired during transmission of the parasite to the mosquito vector. Further in-depth analysis of these mutants using a cross fertilisation approach showed that this defect is limited to male gamete differentiation (exflagellation) and formation since Δ*cdc20* macrogametocytes are fully capable of cross fertilization with microgametes from donor strains. Hence, CDC20 has an essential function for the transition of male gametocytes to gametes. Gametogenesis in *Plasmodium* involves three rounds of mitotic division in male gametocytes resulting in eight gametes [24]–[26], [50]. We have previously shown that CDPK4 is involved in cell cycle progression to S phase and MAP2 may be essential for replication and mitosis to be completed before cytokinesis commences [28], [32], [33] (although it is important to note that MAP2 is essential for asexual development in *P. falciparum* [51], so there may be species-specific differences in the roles of different kinases). As *cdc20* mRNA levels are up regulated in both Δ*cdpk4* and Δ*map2* mutants, this suggests that CDC20 may be interlinked with these kinases and orchestrates the process of male gametogenesis and is perhaps up-regulated to compensate for the loss of these two kinases, but this suggestion requires further investigation. The *cdc20* deletion mutants formed axonemes and mitotic spindles but failed to undergo karyokinesis or cytokinesis and also did not form motile, flagellar gametes, a phenotype similar to what we have observed with *map2* deletion mutants. The requirement for CDC20 during karyokinesis is consistent with the known function of CDC20 and CDH1 in other systems [7]. As described earlier, CDC20 is active during early mitosis in other cells and its up-regulation in gametocytes suggests that it has an essential role in the multiple rounds of DNA replication and the chromosome separation specifically associated with this process. However, mutant *cdc20* parasites do not arrest during asexual proliferation and this suggests that *Plasmodium* CDC20 is specifically required for microgametogenesis.

**Figure 8:**
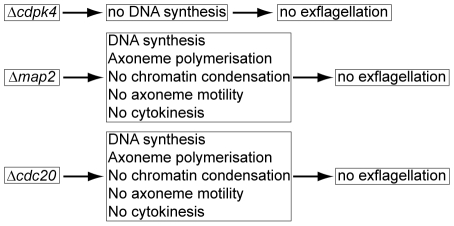
Summary of phenotypes in mutants of *cdpk4, map2* and *cdc20*. *Cdpk4* mutants have been shown to arrest DNA synthesis after activation, whereas *cdc20* mutants show a similar phenotype to *map2* mutants.

Functional studies in human systems have shown that a deficiency of CDH1 results in delayed mitotic exit as well as an accumulation of mitotic errors and difficulty in completion of cytokinesis [52], [53], similar to what is observed in our *cdc20* and *map2* mutants. Therefore we suggest that CDC20 in *Plasmodium* fulfils the function of both CDC20 and CDH1. Moreover, loss of *cdc20* results in arrest during metaphase to anaphase transition [12], [54], [55], with sister chromatids failing to form. How the single CDC20 protein may fulfil the roles of both CDC20 and CDH1 requires further investigation. Our ultrastructure studies for both Δ*cdc20* and Δ*map2* lines, reported for the first time to our knowledge; show that these mutants have a similar arrest in cytokinesis and karyokinesis detected by EM, with defects in nuclear spindle/kinetochore movement and chromatin condensation, confirming our initial light microscopy observation of Δ*map2*. Unlike the Δ*map2* line, we never observed any exflagellation in the Δ*cdc20* line. As suggested before [56], classical spindle checkpoints are not present in *Plasmodium* since blockage of microtubule organisation does not appear to block DNA synthesis. Therefore, MAP2 and CDC20 may be involved in a critical cell cycle checkpoint during microgametogenesis that controls DNA replication and mitosis, prior to karyokinesis and cytokinesis and is summarised in Figure 8.

DNA replication in the Δ*cdc20* line was similar to that in the Δ*map2* line, with both mutants undergoing octoploidy 8 mpa, but not undergoing karyokinesis. As a result, we analysed whether phosphorylation of CDC20 could be involved in mitotic progression during microgametogenesis. In other systems, phosphorylation of CDC20 can be achieved by BUB1, CDK1, MAPK [57], [58] and also NEK2 [59], another protein kinase required for zygote development in *Plasmodium* [60] and this modification is an essential step for CDC20 inhibition by the SAC [61], [62]. Here, we have shown that CDC20 is more phosphorylated in activated gametocytes and ookinetes (i.e. sexual stages) compared to schizont (asexual stages), which suggests that phosphorylation of CDC20 may be a possible mechanism involved in gametogenesis.

Interestingly, the global phosphorylation profile of Δ*cdc20* parasites suggests that CDC20 regulates the phosphorylation of specific proteins within the gametocytes. The proteins that are regulated by CDC20 are; however, largely different from those that appear to be regulated by MAP2. This would suggest that at the level of phosphorylation CDC20 and MAP2 regulate different pathways. It would be interesting in future studies to dissect out the proteins regulated by MAP2 and CDC20 and in this way build a network of phospho-proteins that regulate male gametogenesis.

In conclusion, this study identified significant differences in the control of mitosis during asexual development compared to microgametogenesis in the malaria parasite. We have also shown that CDC20 and MAP2 may play independent but essential roles in the mitotic division associated with microgametogenesis but are not essential for mitosis during asexual stages in the malaria parasite.

## Materials and Methods

### Ethics statement

All animal work has passed an ethical review process and was approved by the United Kingdom Home Office. Work was carried out in accordance with the United Kingdom ‘Animals (Scientific Procedures) Act 1986’ and in compliance with ‘European Directive 86/609/EEC’ for the protection of animals used for experimental purposes. The permit number for the project licence is 40/3344.

### Animals

Either Tuck's Original (TO) (Harlan) or CD1 (CRUK) outbred mice were used for all experiments.

### Generation of transgenic parasites

The targeting vector for *cdc20* was constructed using the pBS-DHFR cassette, in which polylinker sites flank a *Toxoplasma gondii dhfr/ts* expression cassette conveying resistance to pyrimethamine. PCR primers N10-1 (5′-CCCCGGGCCCGAGCTGTCTACTGCTCTGGTAAAGCC-3′) and N10-2 (5′-GGGGAAGCTTCATTATTCTGGATCATAGCTCTC-3′) were used to generate a 452 base pair (bp) fragment 5′ upstream sequence of *Pbcdc20* from genomic DNA, which was inserted into *Apa*I and *Hind*III restriction sites upstream of the *dhfr/ts* cassette of pBS-DHFR. A 579 bp fragment generated with primers N10-3 (5′-CCCCGAATTCGGAACTTCTCTTGTTTCTGGATCTCC-3′) and N10-4 (5′-GGGGTCTAGAGCATGCTAATTAGCTTCACATCCG-3′) from the 3′ flanking region of *Pbcdc20* was then inserted downstream of the *dhfr/ts* cassette using *Eco*RI and *Xba*I restriction sites. The linear targeting sequence was released using *Apa*I/*Xba*I. For GFP-tagging by single homologous recombination and generation of the plasmid for episomal expression, a 2435 bp region of *Pbcdc20* starting 812 bp upstream of the start codon and omitting the stop codon was amplified using primers T36-1 (5′-CCCCGGTACCCTTATTTATGAAAACGATTATAAGG-3′) and T36-2 (5′-CCCCGGGCCCCCTGATTATTTCATAATAATTTTCAAAGGG-3′), producing an amplicon 2435 bp in length. This was then inserted upstream of the *gfp* sequence in the p277 vector using *Kpn*I and *Apa*I restriction sites. The p277 vector contains the human *dhfr* cassette, also conveying resistance to pyrimethamine. Before transfection, the sequence was linearised using *Hind*III and *P. berghei* ANKA line 2.34 was then transfected by electroporation [36]. Briefly, electroporated parasites were mixed immediately with 200 µl of reticulocyte-rich blood from a phenylhydrazine (Sigma) treated, naïve mouse and incubated at 37°C for 30 minutes and then injected intraperitoneally. From day 1 post infection pyrimethamine (7 mg/ml) (Sigma) was supplied in the drinking water for four days. Mice were monitored for 15 days and drug selection repeated after passage to a second mouse, with resistant parasites used for cloning by limiting dilution and genotyping.

### Genotypic analysis of mutants

Chromosomes of wild type and gene knockout parasites were separated by pulsed field gel electrophoresis (PFGE) on a CHEF DR III (Bio-Rad) using a linear ramp of 60–500 s for 72 hr at 4 V/cm. Gels were blotted and hybridized with a probe recognizing both the resistance cassette in the targeting vector and, more weakly, the 3′-untranslated region (UTR) of the *P. berghei dhfr/ts* locus on chromosome 7. For the gene knockout parasites, two diagnostic PCR reactions were used as illustrated in Figure S2. Primer 1 (INT N10, 5′-GTGTGCAATTTGGGAATTTAGCTAG-3′) and primer 2 (ol248, 5′-GATGTGTTATGTGATTAATTCATACAC-3′) were used to determine correct integration of the selectable marker at the targeted locus. Primers 3 (N10 KO1, 5′- GATAATAATTGGAATAGTCATT-3′) and 4 (N10 KO2, 5′- TTACATGTATAACTATTCCA-3′) were used to verify deletion of the target gene. Having confirmed integration, genomic DNA from wild type and mutant parasites was digested with *Hind*III and the fragments were separated on a 0.8% agarose gel, blotted onto a nylon membrane (GE Healthcare), and probed with a PCR fragment homologous to the *P. berghei* genomic DNA just outside of the targeted region.

For the C-fusion GFP tagging parasites, one diagnostic PCR reaction was also used as illustrated in Figure S2. Primer 1 (INT T36, 5′- CATTCCAAACTAGTATTATAAAATTTGTTG -3′) and primer 2 (ol492, 5′- ACGCTGAACTTGTGGCCG-3′) were used to determine correct integration of the *gfp* sequence at the targeted locus. Having confirmed correct integration, genomic DNA from wild type and transgenic parasites was digested with *EcoR*I and the fragments were separated on a 0.8% agarose gel, blotted onto a nylon membrane, and probed with a PCR fragment homologous to the *P. berghei* genomic *cdc20* sequence using the Amersham ECL Direct Nucleic Acid Labelling and Detection kit (GE Healthcare). Parasites were also visualized on a Zeiss AxioImager M2 (Carl Zeiss, Inc) microscope fitted with an AxioCam ICc1 digital camera (Carl Zeiss, Inc) and analysed by Western blot to confirm GFP expression as described.

### Western blotting

Western blot analysis was performed on cell lysates prepared by re-suspending parasite pellets in a 1∶1 ratio of PBS containing Protease inhibitor (Roche) and Laemmli sample buffer, boiling and separating on a 12% SDS-polyacrylamide gel. Samples were subsequently transferred to nitrocellulose membranes (Amersham Biosciences) and immunoblotting performed using the Western Breeze Chemiluminescent Anti-Rabbit kit (Invitrogen) and anti-GFP polyclonal antibody (Invitrogen), according to the manufacturer's instructions.

### Alignment and phylogenetic analysis

The protein sequences of the highly conserved WD domain from CDC20 and CDH1 homologues from a range of eukaryotes were downloaded from NCBI. ClustalW was used to align the sequences and construct a phylogenetic tree.

### Phenotypic analysis

Phenotypic screening of *cdc20* mutants was performed as previously described [20], [37]. Briefly, asexual proliferation and gametocytogenesis were analysed using blood smears. Gamete activation, zygote formation and ookinete conversion rates were monitored by *in vitro* cultures using a marker for the surface antigen P28 as previously described [37], [38]. Hoechst 33342 was used to stain parasite nuclei. Stained cells were analysed on a Zeiss AxioImager M2 microscope (Carl Zeiss, Inc) fitted with an AxioCam ICc1 digital camera (Carl Zeiss, Inc). For mosquito transmission triplicate sets of 50–100 *Anopheles stephensi* mosquitoes were allowed to feed on anaesthetized infected mice on days 4 to 5 following blood infection for 20 min at 20°C. Guts were analysed 14 and 21 days post infection for production of oocysts and sporulating oocysts respectively.

### Ookinete conversion assay

Parasite-infected blood was re-suspended in ookinete medium as previously described [32], [60]. After 24 hours, samples were re-suspended in ookinete medium containing Hoechst DNA dye and anti-P28 Cy3-conjugated 13.1 antibody [32], [37] and examined with the Zeiss AxioImager M2 microscope fitted with an AxioCam ICc1 digital camera (Carl Zeiss, Inc). The percentage of ookinetes to all 13.1-positive cells (unfertilised macrogametes (round cells) and ookinetes) was then calculated.

### Immunocytochemistry and analysis of DNA content

Gametocytes in parasite-infected blood (as described above) were activated in ookinete medium, resuspended in 4% paraformaldehyde (PFA) (Sigma) diluted in microtubule stabilizing buffer (MTSB) [32] and added to poly-L-lysine coated slides. Immunocytochemistry was performed on the fixed parasite material using primary mouse monoclonal anti-alpha tubulin antibody (Sigma, used at 1 in 500). Secondary antibody was Alexa 547 conjugated anti-mouse IgG (Molecular probes, used at 1 in 1000). The slides were then mounted in Vectashield with DAPI (Vector Labs). Parasites were visualized on a Zeiss AxioImager M2 microscope (Carl Zeiss, Inc) fitted with an AxioCam ICc1 digital camera (Carl Zeiss, Inc).

To measure nuclear DNA content of activated microgametocytes by direct immunofluorescence, images of parasites fixed and stained as above were analyzed using the ImageJ software (version 1.44) (National Institute of Health) as previously described [32].

To confirm nuclear DNA content of activated microgametocytes by FACS, purified gametocytes were transferred to standard ookinete culture medium for activation of gamete formation. At 8 mins after activation cells were pelleted by centrifugation (5 sec; 10,000 rpm), fixed in 0.25% glutaraldehyde/PBS solution and stained with 2 µM Hoechst-33258. The Hoechst-fluorescence intensity (DNA content) of the gametocytes was analyzed by FACS using a LSR-II flow cytometer (Becton Dickinston). Cells were analyzed at room temperature with the following filters (parameters/thresholds): UB 440/40 (Hoechst) (400/5000); FSC (250/2000); SSC (200/5000). The cells for analysis were selected on size by gating on FSC and SSC. A total of 10,000–500,000 cells were analyzed per sample and all measurements were performed on triplicate samples. To determine the Hoechst-fluorescence intensity (DNA content) from the populations of activated female and male gametocytes, gates were set as in [63]. Data processing and analysis was performed using the program FlowJo (http://www.flowjo.com).

### Electron microscopy

Samples of wild type, *cdc20* mutant and *map2* mutant microgametocytes cultured as described above were fixed in 4% glutaraldehyde in 0.1 M phosphate buffer and processed for routine electron microscopy as described previously [64]. Briefly, samples were post fixed in osmium tetroxide, treated *en bloc* with uranyl acetate, dehydrated and embedded in Spurr's epoxy resin. Thin sections were stained with uranyl acetate and lead citrate prior to examination in a JEOL12EX electron microscope (Jeol AB).

Quantitation of the nuclear features observed by electron microscopy was carried out at 15 and 30 minutes. This was based on the examination of 100 microgametocytes identified by axoneme formation at each time point. The features identified were ^1^nuclei with no specific features in the plan of section, ^2^early stage exhibiting nuclear poles with spindle microtubules and kinetochores, ^3^mid stage with nuclear pole but no attached kinetochores, and ^4^late stages with the nucleus exhibiting areas of condensed chromatin.

### Purification of gametocytes and ookinetes

Purification of gametocytes was achieved using a modified protocol from [65]. Briefly, mice were treated by intra-peritoneal injection of 0.2 ml of phenylhydrazine (6 mg/ml) (Sigma) in PBS to encourage reticulocyte formation four days prior to infection with parasites. Day four post infection (p.i.) mice were treated with sulfadiazine (Sigma) at 20 mg/L in their drinking water for two days to eliminate asexual blood stage parasites. On day six p.i. mice were bled by cardiac puncture into heparin and gametocytes separated from uninfected erythrocytes on a NycoDenz gradient made up from 48% NycoDenz (27.6% w/v NycoDenz in 5 mM Tris-HCl, pH 7.20, 3 mM KCl, 0.3 mM EDTA) and coelenterazine loading buffer (CLB), containing PBS, 20 mM HEPES, 20 mM Glucose, 4 mM sodium bicarbonate, 1 mM EGTA, 0.1% w/v bovine serum albumin, pH 7.25. Gametocytes were harvested from the interface and washed twice in RPMI 1640 ready for activation of gamete formation. Blood from day 5 pi mice were cultured for 24 hrs at 20°C for ookinetes as described above. Ookinetes were purified on a 63% NycoDenz gradient and harvested from the interface, washed and labelled.

### Quantitative RT-PCR

Parasites were purified as described and frozen in Trizol (Sigma) prior to RNA extraction. RNA was isolated according to manufacturer's instructions. Isolated RNA was treated with DNase I (Promega) and used in reverse transcription reactions (SuperScript III Reverse Transcription kit, Invitrogen) from 1 µg of total RNA.

Gene expression was quantified by SYBR green PCR using Fast mastermix on an ABI 7500 QPCR System (Applied Biosystems). Primers were designed using the PerlPrimer software program [66] to be 18–22 bps in length, with 30–60% GC content, to amplify a region 50–150 bp long and when possible, to bind within 600 bp of the 3′ end of the genes of interest. Primer efficiencies were all between 90–110%, with qRT-PCR resulting in no detectable primer dimers, as determined by dissociation curves. cDNA was diluted 1∶20 with DEPC-treated water before use. Reactions consisted of 3.6 µl of diluted cDNA, 5 µl SYBR green fast mastermix (Applied Biosystems), 0.2 µl each of forward and reverse primer and 1 µl of DEPC water. Cycling conditions were: 95°C for 20 sec followed by 40 cycles of 95°C, 3 secs, and 60°C, 30 secs, followed by dissociation curve. Three biological replicates, with three technical replicates from each biological replicate were performed for each assayed gene. Endogenous gene expression was determined using the comparative cycle threshold method [67], whereas relative quantification in mutant lines was determined using the Pfaffl method [68]. Both methods used *hsp70* (PBANKA_081890) (forward, 5′-GTATTATTAATGAxACCCACCGCT-3′; reverse, 5′-GAAACATCAAATGTACCAxCCTCC-3′) and *arginyl-tRNA synthetase* (PBANKA_143420) (forward, 5′-TTGATTCATGTTGGATTTGGCT-3′; reverse, 5′-ATCCTTCTTTGCCCTTTCAG-3′) as reference genes. *cdc20* primers were: forward, 5′-ATGTTTGGTAACTATTTGGCGG-3′; reverse, 5′-ATCCCATATTTCTACTGCACCA-3′. *map2* (PBANKA_093370): forward, 5′-AATGAAGAACCAGGGCCA-3′; reverse, 5′-ACCATCTAGTAACTACATGGCT-3′. *cdpk4* (PBANKA_061520): forward, 5′-AAATGTTGATGTACACAAGTGC-3′; reverse, 5′-ATGTTCTAATGCATCTCTCTTGCT-3′

### CDC20 phosphorylation in vivo

Blood aliquots from infected mice were incubated overnight, from which schizonts and ookinetes were purified by using Nycodenz protocols as described previously [36], [65]. Gametocytes were purified and activated for 25 min at 20°C in ookinete medium as described above. Schizonts, activated gametocytes and ookinetes were then washed in phosphate-free Kreb's buffer and metabolically labelled with 3–5MBq ^32^P-orthophosphate in phosphate-free Kreb's buffer for 30 min at 20°C. After two washes in phosphate-free Kreb's buffer, the labelled parasites were lysed for 30 min at 4°C in lysis buffer (10 mM Tris pH 7.5, 150 mM NaCl, 0.5 mM EDTA, 0.5% NP-40) supplemented with protease and phosphatase inhibitors (Roche), the resulting lysate was centrifuged at 20,000×g for 5 min and the supernatant collected. GFP-tagged CDC20 proteins were then immunoprecipitated using GFP-TRAP beads (ChromoTek). The immunoprecipitated proteins were then resuspended in Laemmli sample buffer and separated by SDS-PAGE. ^32^P-labelled proteins were visualized using a phosphorimager (Molecular Dynamics) and GFP-tagged proteins analysed by Western Blot as described above, using an anti-GFP polyclonal antibody (Invitrogen). The relative CDC20-GFP phosphorylation levels in activated gametocytes and ookinetes with respect to schizonts were obtained by taking the normalized ratio between the intensity of the phosphorylation signal from the phosphorimager and the intensity of the GFP immunoreactive signal from the corresponding Western Blot by using the ImageJ software (National Institute of Health).

### Metabolic labelling for phosphorylation profile

Gametocytes from wild type, *cdc20* and *map2* mutant parasites were purified by using a Nycodenz protocol as described above from the blood of infected mice. Purified gametocytes were placed for 25 minutes in ookinete medium at 20°C to activate both male and female gametocytes to form gametes. For metabolic labelling, the parasites were washed once with 1 ml of phosphate-free Kreb's buffer: 118 mM NaCl, 4.7 mM KCl, 4.2 mM NaHCO_3_, 1.2 mM MgSO_4_(2H_2_O), 11.7 mM glucose, 10 mM HEPES, 1.3 mM CaCl_2_(2H_2_O), pH 7.4 and resuspended in 500 µl of the same buffer. 20–25 µl ^32^P-orthophosphate (7–9.25MBq) was added to the suspension and incubated at 37°C for 30 min. The labelled parasites were then lysed in lysis buffer: 50 mM Tris, 0.5 mM EDTA, 5% β-glycerolphosphate, pH 7.6, supplemented with protease/phosphatase inhibitors (Roche) and 1% NP-40. Following incubation on ice for 10 min, the samples were centrifuged 3 min at 20000×g and the supernatants were collected for further fractionation. Fractionation was carried out on an AKTA chromatographer (Amersham Pharmacia Biotec) using Resource Q (Amersham Pharmacia Biotec) anion-exchange column (matrix volume 1 ml). The proteins were eluted using a linear gradient of 0–1.0 M NaCl in running buffer: 10 mM Tris, 5 mM EDTA and 20 mM β-glycerolphosphate, pH 7.4. Fractions (1 ml) were collected and analysed further by resolution on SDS-PAGE gels. ^32^P-labelled proteins were visualised by autoradiography.

### Statistical analyses

All statistical analyses were performed using GraphPad Prism (GraphPad Software). For ookinete conversion rates, non-parametric t-tests were used. For relative quantification of qRT-PCR reactions, two-way ANOVA was performed.

## Supporting Information

Figure S1 **Clustal W alignments used for phylogenetic analyses.** Multiple amino-acid sequence alignments of the conserved WD repeat domains from different species were performed using the Clustal W program. Accession numbers used for alignments were: *S.cerevisiae* Cdc20 (NP_011399.1), *S.pombe* Slp1 (NP_593161.1), *L.major* Cdc20 (XP_001683689), *L.infantum* Cdc20 (XP_003392580.1), *L.braziliensis* Cdc20 (XP_001565442.1), *T.brucei* Cdc20 (XP_847480.1), *T.cruzi* Cdc20 (XP_819329.1), *S.cerevisiae* Cdh1 (NP_011512.1), *S.pombe* Srw1 (CAB59693), *H.sapiens* Cdh1 (NP_057347.2), *M.musculus* Cdh1 (NP_062731.1), *D.rerio* fizzy-related (NP_956547.1), *D.melanogaster* fizzy-related (CAA74575.1), *C.elegans* fzr-1 (NP_496075.1), *C.briggsae* Cdh1 (XP_002648545.1), *A.thaliana* Cdh1.1 (NP_192929.2), *A.thaliana* Cdh1.2 (NP_194022.3), *A.thaliana* Cdh1.3 (NP_196888.2), *V.carteri* Cdc20 (XP_002950513.1), *C.hominis* Cdc20 (XP_665894.1), *C.parvum* Cdc20 (XP_628181.1), *C.muris* Cdc20 (XP_002142595.1), *H.sapiens* Cdc20 (NP_001246.2), *M.musculus* Cdc20 (NP_075712.2), *D.rerio* Cdc20 (NP_998245.1), *D.melanogaster* fizzy (NP_477501.1), *A.thaliana* Cdc20.1 (NP_195053.1), *A.thaliana* Cdc20.2 (AEE86199.1), *A.thaliana* Cdc20.3 (AED93647.1), *A.thaliana* Cdc20.4 (AED93621.1), *A.thaliana* Cdc20.5 (AED93702.1), *Micromonas* Cdc20 (XP_002502587.1), *P.yoelii* Cdc20 (XP_728399.1), *P.berghei* Cdc20 (XP_679699.1), *P.chaubaudi* Cdc20 (XP_743667.1), *P.falciparum* Cdc20 (XP_001347545.1), *P.knowlesi* Cdc20 (XP_002261784.1), *P.vivax* Cdc20 (XP_001608503.1), *S.cerevisiae* Ama1 (NP_011741.3).
(PDF)

Figure S2 ***gfp*** **tagging and targeted disruption of the** ***Pbcdc20*** **locus.** A. Schematic representation of the gene targeting strategy used for gene tagging the endogenous locus with *gfp* via single homologous recombination. Primers 1+2 used for diagnostic PCR are indicated, as well as the *EcoR*I site used for Southern blotting. Probe location used for detection by Southern blotting is indicated. B. Diagnostic PCR confirming successful integration of the tagging sequence. C. Southern blot analysis of *EcoR*I digested T36 genomic DNA using the 3′ UTR of the targeting construct as a probe. Band sizes for CDC20-GFP (*tag*) and wild-type (*wt*) are indicated. D. Western blot analysis using an anti-GFP antibody against control wild-type-GFP (wt) and transgenic (tag) activated gametocytes showing bands of expected sizes of 29 kDa for wild-type-GFP and 92 kDa for PbCDC20-GFP. E. Schematic representation of the gene targeting strategy used for gene disruption via double homologous recombination. Primers 1–4 used for diagnostic PCR are indicated, as well as the *Hind*III digestion site used for Southern blotting. Probe location used for detection by Southern blotting is indicated. F. Diagnostic PCR confirming successful integration of the disruption sequence of *cdc20* in mutants N10 clone 7 (*cl7*) and N10 clone 9 (*cl9*). Primers 1+2 were used to verify successful integration at the correct locus. Primers 3+4 were used to confirm loss of the endogenous gene. G. Southern blot analysis of *Hin*dIII digested N10 clone 7 genomic DNA using the 5′ UTR of the targeting construct as a probe. Band sizes for N10 clone 7 (*cl7*) and wild-type (*wt*) are indicated. H. Pulse-field gel electrophoresis blot hybridised with *Pb* 3′UTR which detects the endogenous chromosome 7 locus and disrupted locus on chromosome 5 in both clones. I. Bar graph showing relative expression of endogenous *Pbcdc20* in *Δcdc20* mutants using qRT-PCR compared to wild-type. Error bars represent ±SEM, *n = *3 from three separate experiments in both clone 7 and clone 9.
(DOC)

Figure S3 **Episomal expression of PbCDC20-GFP.** Episomal expression of CDC20-GFP throughout the life-cycle was shown to co-localise with Hoechst staining at all stages with addition cytoplasmic expression in ookinetes. High GFP intensity was observed at all stages. Bar = 5 µm. Female gametes (*), zygotes (z) and ookinetes (arrow) are indicated.
(TIF)

## References

[1] Morgan DO (1999). Regulation of the APC and the exit from mitosis.. Nat Cell Biol.

[2] Dawson IA, Roth S, Artavanis-Tsakonas S (1995). The Drosophila cell cycle gene fizzy is required for normal degradation of cyclins A and B during mitosis and has homology to the CDC20 gene of Saccharomyces cerevisiae.. J Cell Biol.

[3] Weinstein J, Jacobsen FW, Hsu-Chen J, Wu T, Baum LG (1994). A nove mammalian protein, p55CDC, present in dividing cells is associated with protein kinase activity and has homology to the Saccharomyces cerevisiae cell division cycle proteins Cdc20 and Cdc4.. Mol Cell Biol.

[4] Wada Y, Kitamoto K, Kanbe T, Tanaka K, Anraku Y (1990). The SLP1 gene of Saccharomyces cerevisiae is essential for vacuolar morphogenesis and function.. Mol Cell Biol.

[5] Schwab M, Lutum AS, Seufert W (1997). Yeast Hct1 is a regulator of Clb2 cyclin proteolysis.. Cell.

[6] Sigrist SJ, Lehner CF (1997). Drosophila fizzy-related down-regulates mitotic cyclins and is required for cell proliferation arrest and entry into endocycles.. Cell.

[7] Kramer ER, Scheuringer N, Podtelejnikov AV, Mann M, Peters JM (2000). Mitotic regulation of the APC activator proteins CDC20 and CDH1.. Mol Biol Cell.

[8] Cebolla A, Vinardell JM, Kiss E, Olah B, Roudier F (1999). The mitotic inhibitor ccs52 is required for endoreduplication and ploidy-dependent cell enlargement in plants.. EMBO J.

[9] Yu H (2007). Cdc20: a WD40 activator for a cell cycle degradation machine.. Mol Cell.

[10] Fang G, Yu H, Kirschner MW (1998). Direct binding of CDC20 protein family members activates the anaphase-promoting complex in mitosis and G1.. Mol Cell.

[11] Kraft C, Herzog F, Gieffers C, Mechtler K, Hagting A (2003). Mitotic regulation of the human anaphase-promoting complex by phosphorylation.. EMBO J.

[12] Shirayama M, Toth A, Galova M, Nasmyth K (1999). APC(Cdc20) promotes exit from mitosis by destroying the anaphase inhibitor Pds1 and cyclin Clb5.. Nature.

[13] Glotzer M, Murray AW, Kirschner MW (1991). Cyclin is degraded by the ubiquitin pathway.. Nature.

[14] Crasta K, Lim HH, Giddings TH, Winey M, Surana U (2008). Inactivation of Cdh1 by synergistic action of Cdk1 and polo kinase is necessary for proper assembly of the mitotic spindle.. Nat Cell Biol.

[15] Musacchio A, Salmon ED (2007). The spindle-assembly checkpoint in space and time.. Nat Rev Mol Cell Biol.

[16] Taylor SS, Scott MI, Holland AJ (2004). The spindle checkpoint: a quality control mechanism which ensures accurate chromosome segregation.. Chromosome Res.

[17] De Antoni A, Pearson CG, Cimini D, Canman JC, Sala V (2005). The Mad1/Mad2 complex as a template for Mad2 activation in the spindle assembly checkpoint.. Curr Biol.

[18] Peters JM (2006). The anaphase promoting complex/cyclosome: a machine designed to destroy.. Nat Rev Mol Cell Biol.

[19] Pesin JA, Orr-Weaver TL (2008). Regulation of APC/C activators in mitosis and meiosis.. Annu Rev Cell Dev Biol.

[20] Tewari R, Straschil U, Bateman A, Bohme U, Cherevach I (2010). The systematic functional analysis of Plasmodium protein kinases identifies essential regulators of mosquito transmission.. Cell Host Microbe.

[21] Bannister LH, Sherman IW (2009). Plasmodium.. Encyclopedia of Life Sciences (ELS) Chichester: John Wiley & Sons, Ltd.

[22] Aikawa M, Beaudoin RL (1968). Studies on nuclear division of a malarial parasite under pyrimethamine treatment.. J Cell Biol.

[23] Brooks CF, Francia ME, Gissot M, Croken MM, Kim K (2011). Toxoplasma gondii sequesters centromeres to a specific nuclear region throughout the cell cycle.. Proc Natl Acad Sci U S A.

[24] Sinden RE, Canning EU, Bray RS, Smalley ME (1978). Gametocyte and gamete development in Plasmodium falciparum.. Proc R Soc Lond B Biol Sci.

[25] Sinden RE, Canning EU, Spain B (1976). Gametogenesis and fertilization in Plasmodium yoelii nigeriensis: a transmission electron microscope study.. Proc R Soc Lond B Biol Sci.

[26] Sinden RE, Talman A, Marques SR, Wass MN, Sternberg MJ (2010). The flagellum in malarial parasites.. Curr Opin Microbiol.

[27] Gerald N, Mahajan B, Kumar S (2011). Mitosis in the human malaria parasite Plasmodium falciparum.. Eukaryot Cell.

[28] Billker O, Dechamps S, Tewari R, Wenig G, Franke-Fayard B (2004). Calcium and a calcium-dependent protein kinase regulate gamete formation and mosquito transmission in a malaria parasite.. Cell.

[29] Billker O, Lindo V, Panico M, Etienne AE, Paxton T (1998). Identification of xanthurenic acid as the putative inducer of malaria development in the mosquito.. Nature.

[30] Billker O, Shaw MK, Margos G, Sinden RE (1997). The roles of temperature, pH and mosquito factors as triggers of male and female gametogenesis of Plasmodium berghei in vitro.. Parasitology.

[31] Khan SM, Franke-Fayard B, Mair GR, Lasonder E, Janse CJ (2005). Proteome analysis of separated male and female gametocytes reveals novel sex-specific Plasmodium biology.. Cell.

[32] Tewari R, Dorin D, Moon R, Doerig C, Billker O (2005). An atypical mitogen-activated protein kinase controls cytokinesis and flagellar motility during male gamete formation in a malaria parasite.. Mol Microbiol.

[33] Rangarajan R, Bei AK, Jethwaney D, Maldonado P, Dorin D (2005). A mitogen-activated protein kinase regulates male gametogenesis and transmission of the malaria parasite Plasmodium berghei.. EMBO Rep.

[34] Kramer ER, Gieffers C, Holzl G, Hengstschlager M, Peters JM (1998). Activation of the human anaphase-promoting complex by proteins of the CDC20/Fizzy family.. Curr Biol.

[35] Listovsky T, Brandeis M, Zilberstein D (2011). Leishmania express a functional Cdc20 homologue.. Biochem Biophys Res Commun.

[36] Janse CJ, Franke-Fayard B, Mair GR, Ramesar J, Thiel C (2006). High efficiency transfection of Plasmodium berghei facilitates novel selection procedures.. Mol Biochem Parasitol.

[37] Liu Y, Tewari R, Ning J, Blagborough AM, Garbom S (2008). The conserved plant sterility gene HAP2 functions after attachment of fusogenic membranes in Chlamydomonas and Plasmodium gametes.. Genes Dev.

[38] Reininger L, Billker O, Tewari R, Mukhopadhyay A, Fennell C (2005). A NIMA-related protein kinase is essential for completion of the sexual cycle of malaria parasites.. J Biol Chem.

[39] Dulla K, Daub H, Hornberger R, Nigg EA, Korner R (2010). Quantitative site-specific phosphorylation dynamics of human protein kinases during mitotic progression.. Mol Cell Proteomics.

[40] Solyakov L, Cain K, Tracey BM, Jukes R, Riley AM (2004). Regulation of casein kinase-2 (CK2) activity by inositol phosphates.. J Biol Chem.

[41] Kevei Z, Baloban M, Da Ines O, Tiricz H, Kroll A (2011). Conserved CDC20 Cell Cycle Functions Are Carried out by Two of the Five Isoforms in Arabidopsis thaliana.. PLoS One.

[42] Ponts N, Saraf A, Chung DW, Harris A, Prudhomme J (2011). Unraveling the ubiquitome of the human malaria parasite.. J Biol Chem.

[43] Pfleger CM, Kirschner MW (2000). The KEN box: an APC recognition signal distinct from the D box targeted by Cdh1.. Genes Dev.

[44] Rape M, Kirschner MW (2004). Autonomous regulation of the anaphase-promoting complex couples mitosis to S-phase entry.. Nature.

[45] Rape M, Reddy SK, Kirschner MW (2006). The processivity of multiubiquitination by the APC determines the order of substrate degradation.. Cell.

[46] Hall N, Karras M, Raine JD, Carlton JM, Kooij TW (2005). A comprehensive survey of the Plasmodium life cycle by genomic, transcriptomic, and proteomic analyses.. Science.

[47] Kallio MJ, Beardmore VA, Weinstein J, Gorbsky GJ (2002). Rapid microtubule-independent dynamics of Cdc20 at kinetochores and centrosomes in mammalian cells.. J Cell Biol.

[48] Sironi L, Melixetian M, Faretta M, Prosperini E, Helin K (2001). Mad2 binding to Mad1 and Cdc20, rather than oligomerization, is required for the spindle checkpoint.. EMBO J.

[49] Le Roch KG, Zhou Y, Blair PL, Grainger M, Moch JK (2003). Discovery of gene function by expression profiling of the malaria parasite life cycle.. Science.

[50] Sinden RE (1991). Mitosis and meiosis in malarial parasites.. Acta Leiden.

[51] Dorin-Semblat D, Quashie N, Halbert J, Sicard A, Doerig C (2007). Functional characterization of both MAP kinases of the human malaria parasite Plasmodium falciparum by reverse genetics.. Mol Microbiol.

[52] Garcia-Higuera I, Manchado E, Dubus P, Canamero M, Mendez J (2008). Genomic stability and tumour suppression by the APC/C cofactor Cdh1.. Nat Cell Biol.

[53] Li M, Shin YH, Hou L, Huang X, Wei Z (2008). The adaptor protein of the anaphase promoting complex Cdh1 is essential in maintaining replicative lifespan and in learning and memory.. Nat Cell Biol.

[54] Li M, York JP, Zhang P (2007). Loss of Cdc20 causes a securin-dependent metaphase arrest in two-cell mouse embryos.. Mol Cell Biol.

[55] Lim HH, Goh PY, Surana U (1998). Cdc20 is essential for the cyclosome-mediated proteolysis of both Pds1 and Clb2 during M phase in budding yeast.. Curr Biol.

[56] Billker O, Shaw MK, Jones IW, Ley SV, Mordue AJ (2002). Azadirachtin disrupts formation of organised microtubule arrays during microgametogenesis of Plasmodium berghei.. J Eukaryot Microbiol.

[57] Tang Z, Shu H, Oncel D, Chen S, Yu H (2004). Phosphorylation of Cdc20 by Bub1 provides a catalytic mechanism for APC/C inhibition by the spindle checkpoint.. Mol Cell.

[58] D'Angiolella V, Mari C, Nocera D, Rametti L, Grieco D (2003). The spindle checkpoint requires cyclin-dependent kinase activity.. Genes Dev.

[59] Liu Q, Hirohashi Y, Du X, Greene MI, Wang Q (2010). Nek2 targets the mitotic checkpoint proteins Mad2 and Cdc20: a mechanism for aneuploidy in cancer.. Exp Mol Pathol.

[60] Reininger L, Tewari R, Fennell C, Holland Z, Goldring D (2009). An essential role for the Plasmodium Nek-2 Nima-related protein kinase in the sexual development of malaria parasites.. J Biol Chem.

[61] Chung E, Chen RH (2003). Phosphorylation of Cdc20 is required for its inhibition by the spindle checkpoint.. Nat Cell Biol.

[62] Yudkovsky Y, Shteinberg M, Listovsky T, Brandeis M, Hershko A (2000). Phosphorylation of Cdc20/fizzy negatively regulates the mammalian cyclosome/APC in the mitotic checkpoint.. Biochem Biophys Res Commun.

[63] Laurentino EC, Taylor S, Mair GR, Lasonder E, Bartfai R (2011). Experimentally controlled downregulation of the histone chaperone FACT in Plasmodium berghei reveals that it is critical to male gamete fertility.. Cell Microbiol.

[64] Ferguson DJ, Henriquez FL, Kirisits MJ, Muench SP, Prigge ST (2005). Maternal inheritance and stage-specific variation of the apicoplast in Toxoplasma gondii during development in the intermediate and definitive host.. Eukaryot Cell.

[65] Beetsma AL, van de Wiel TJ, Sauerwein RW, Eling WM (1998). Plasmodium berghei ANKA: purification of large numbers of infectious gametocytes.. Exp Parasitol.

[66] Marshall OJ (2004). PerlPrimer: cross-platform, graphical primer design for standard, bisulphite and real-time PCR.. Bioinformatics.

[67] Schmittgen TD, Livak KJ (2008). Analyzing real-time PCR data by the comparative C(T) method.. Nat Protoc.

[68] Pfaffl MW (2001). A new mathematical model for relative quantification in real-time RT-PCR.. Nucleic Acids Res.

[69] Janse CJ, van der Klooster PF, van der Kaay HJ, van der Ploeg M, Overdulve JP (1986). DNA synthesis in Plasmodium berghei during asexual and sexual development.. Mol Biochem Parasitol.

