# Correction: A Putative Homologue of CDC20/CDH1 in the Malaria Parasite Is Essential for Male Gamete Development

**DOI:** 10.1371/annotation/ef70d427-0816-4a63-aeaf-874b734793b0

**Published:** 2012-03-28

**Authors:** David S. Guttery, David J. P. Ferguson, Benoit Poulin, Zhengyao Xu, Ursula Straschil, Onny Klop, Lev Solyakov, Sara M. Sandrini, Declan Brady, Conrad A. Nieduszynski, Chris J. Janse, Anthony A. Holder, Andrew B. Tobin, Rita Tewari

Figure S2 is a duplicate of Figure S1. Please view the correct Figure S2 file here: 
