# Supplementary material for: A Putative Homologue of CDC20/CDH1 in the Malaria Parasite Is Essential for Male Gamete Development
Source: PLoS Pathog. 2012 Feb 23;8(2):e1002554. doi: 10.1371/journal.ppat.1002554 (PMC3285604; doi:10.1371/journal.ppat.1002554)
Supplement: Figure S1 — Clustal W alignments used for phylogenetic analyses. Multiple amino-acid sequence alignments of the conserved WD repeat domains from different species were performed using the Clustal W program. Accession numbers used for alignments were: S.cerevisiae Cdc20 (NP_011399.1), S.pombe Slp1 (NP_593161.1), L.major Cdc20 (XP_001683689), L.infantum Cdc20 (XP_003392580.1), L.braziliensis Cdc20 (XP_001565442.1), T.brucei Cdc20 (XP_847480.1), T.cruzi Cdc20 (XP_819329.1), S.cerevisiae Cdh1 (NP_011512.1), S.pombe Srw1 (CAB59693), H.sapiens Cdh1 (NP_057347.2), M.musculus Cdh1 (NP_062731.1), D.rerio fizzy-related (NP_956547.1), D.melanogaster fizzy-related (CAA74575.1), C.elegans fzr-1 (NP_496075.1), C.briggsae Cdh1 (XP_002648545.1), A.thaliana Cdh1.1 (NP_192929.2), A.thaliana Cdh1.2 (NP_194022.3), A.thaliana Cdh1.3 (NP_196888.2), V.carteri Cdc20 (XP_002950513.1), C.hominis Cdc20 (XP_665894.1), C.parvum Cdc20 (XP_628181.1), C.muris Cdc20 (XP_002142595.1), H.sapiens Cdc20 (NP_001246.2), M.musculus Cdc20 (NP_075712.2), D.rerio Cdc20 (NP_998245.1), D.melanogaster fizzy (NP_477501.1), A.thaliana Cdc20.1 (NP_195053.1), A.thaliana Cdc20.2 (AEE86199.1), A.thaliana Cdc20.3 (AED93647.1), A.thaliana Cdc20.4 (AED93621.1), A.thaliana Cdc20.5 (AED93702.1), Micromonas Cdc20 (XP_002502587.1), P.yoelii Cdc20 (XP_728399.1), P.berghei Cdc20 (XP_679699.1), P.chaubaudi Cdc20 (XP_743667.1), P.falciparum Cdc20 (XP_001347545.1), P.knowlesi Cdc20 (XP_002261784.1), P.vivax Cdc20 (XP_001608503.1), S.cerevisiae Ama1 (NP_011741.3). (PDF) [file ppat.1002554.s001.pdf]

CLUSTAL 2.1 multiple sequence alignment

```
S._cerevisiae_Cdc20                 ---SKK-NVLAIALDTALY-LWNATTGDVSLLTDFENTT-----------
S._pombe_Slp1                       LDWSNL-NVVAVALERNVY-VWNADSGSVSALAETDESTY----------
L._major_Cdc20                      IDWSATSDVLCVALQNCVY-LWDAKTCGITELPRVVPTGGGLHGDGRSGD
L._infantum_Cdc20                   IDWSATSDVLCVALQNCVY-LWDAKTCGITELPRVVSTGGGLHGDGRSGD
L._braziliensis_Cdc20               IDWSATSDILGVALQNCVY-LWNAKTCDITELPRVVSTGSGMHGEGRSAN
T._brucei_Cdc20                     MDWSAK-DVLAVGLQGSVY-LWYEKTSNIAQLPCQRPANG----------
T._cruzi_Cdc20                      IDWSSK-DILAVGLQGAVY-LWDAKTCNITHLPCQRPPNG----------
S._cerevisiae_Cdh1                  IDWSST-DVLAVALGKSIF-LTDNNTGDVVHLCDTEN-------------
S._pombe_Srw1                       -----------------------------------TD-------------
H._sapiens_Cdh1                     VDWSSL-NVLSVGLGTCVY-LWSACTSQVTRLCDLSV--E----------
M._musculus_Cdh1                    VDWSSL-NVLSVGLGTCVY-LWSACTSQVTRLCDLSV--E----------
D._rerio_fizzy-related              VDWSSL-NVLSVGLGTCVY-LWSACTSQVTRLCDLSV--E----------
D._melanogaster_fizzy-related       VDWSSQ-NVLAVGLGSCVY-LWSACTSQVTRLCDLSP--D----------
C._elegans_fzr-1                    VDWSSQ-NQLSVGLAACVY-LWSATTSQVIKLCDLGQTNE----------
C._briggsae_Cdh1                    VDWSSQ-NQLSVGLSTCVY-LWSATTSQVIKLCDLSASNE----------
A._thaliana_Cdh1.1                  VDWSAQ-NVLAVGLGNCVY-LWNACSSKVTKLCDLGV-------------
A._thaliana_Cdh1.2                  VDWSAQ-NVLAVGLGNCVY-LWNACSSKVTKLCDLGA-------------
A._thaliana_Cdh1.3                  ------------------Y-LWTASNSKVTKLCDLGP-------------
V._carteri_Cdc20                    VDWSSQ-NVLAVGLGTCVY-LWSAMSSTVTKLCDLAP-------------
C._hominis_Cdc20                    --------------------------------------------------
C._parvum_Cdc20                     --------------------------------------------------
C._muris_Cdc20                      VDWSST-NLLAVGLSSSLY-LWNASTSKVTNLMSLPE-------------
H._sapiens_Cdc20                    VDWSSG-NVLAVALDNSVY-LWSASSGDILQLLQMEQPGEY---------
M._musculus_Cdc20                   VDWSSG-NVLAVALDNSVY-LWNAGSGDILQLLQMEQPGDY---------
D._rerio_Cdc20                      -----Q-NVLAVGLANQVY-LWDAGEGDIVLLKKMEDDNEY---------
D._melanogaster_fizzy               MDWSAD-NIVAVALGSCVY-LWNAQTGNIEQLTEFEE-GDY---------
A._thaliana_Cdc20.1                 --------------DHTVY-LWDASTGSTSELVTIDEEKGP---------
A._thaliana_Cdc20.2                 --------------DHTVY-LWDASTGSTSELVTIDEEKGP---------
A._thaliana_Cdc20.3                 -------NVLAIALGDTVY-LWDASSGSTSELVTIDEDKGP---------
A._thaliana_Cdc20.4                 -------NVLAIALGDTVY-LWDASSGSTSELVTIDEDKGP---------
A._thaliana_Cdc20.5                 ----SS-NVLAIALGDTVY-LWDASSGSTYKLVTIDEEEGP---------
Micromonas_Cdc20                    -----------------VY-LWNADSGDIQQLCQTDPNNGDD--------
P._yoelii_Cdc20                     --------------------------------------------------
P._berghei_Cdc20                    --------------------------------------------------
P._chaubaudi_Cdc20                  --------------------------------------------------
P._falciparum_Cdc20                 --------------------------------------------------
P._knowlesi_Cdc20                   --------------------------------------------------
P._vivax_Cdc20                      --------------------------------------------------
S._cerevisiae_Ama1                  HQYLSEKRDLVTCVSFCPYNTYFIVGTKFGRILLYDQK------------


S._cerevisiae_Cdc20                 ---ICSVTWSDDDCHISIGKE--DGNTEIWDVETMSLIRTMRSGLGVRIG
S._pombe_Slp1                       ---VASVKWSHDGSFLSVGLG--NGLVDIYDVESQTKLRTM-AGHQARVG
L._major_Cdc20                      AQLVCGLNWAPDGCHLAVGGH--SGAVEVWDVETQQIVHTYRQ-HADRTV
L._infantum_Cdc20                   AQLVCGLNWAPDGCHLAVGRH--SGAVEVWDVETQQIVHTYRQ-HADRTV
L._braziliensis_Cdc20               AQLVCGLNWAPDGRHLAIGRN--SGAVEVWDVEAQRIVHTYRQ-HADRTV
T._brucei_Cdc20                     --IICGVSWSEDGNHLALGAD--DGSVEIWDVEAERITRRLHH-HTDRVG
T._cruzi_Cdc20                      --IFCGVTWSEDGNLLALGTD--DGSLEIWDVEMQRITRRLYQ-HTDRVG
S._cerevisiae_Cdh1                  --EYTSLSWIGAGSHLAVGQA--NGLVEIYDVMKRKCIRTLSG-HIDRVA
S._pombe_Srw1                       --TVTSLRWVQRGTHLAVGTH--NGSVEIWDAATCKKTRTMSG-HTERVG
H._sapiens_Cdh1                     GDSVTSVGWSERGNLVAVGTH--KGFVQIWDAAAGKKLSMLEG-HTARVG
M._musculus_Cdh1                    GDSVTSVGWSERGNLVAVGTH--KGFVQIWDAAAGKKLSMLEG-HTARVG
D._rerio_fizzy-related              GDSVTSVGWSERGNLVAVGTH--KGFVQIWDATAGKKLFALEG-HTARVG
D._melanogaster_fizzy-related       ANTVTSVSWNERGNTVAVGTH--HGYVTVWDVAANKQINKLNG-HSARVG
C._elegans_fzr-1                    QDQVTSVQWCDKGDLLAVGTS--RGVTQIWDVTTQKKTRELTG-HSSRVG
C._briggsae_Cdh1                    QDQVTSVQWCDKGDLLAVGTN--RGITQIWDVTTQKKIRDLGG-HTSRVG
A._thaliana_Cdh1.1                  DETVCSVGWALRGTHLAIGTS--SGTVQIWDVLRCKNIRTMEG-HRLRVG
A._thaliana_Cdh1.2                  EDSVCSVGWALRGTHLAVGTS--TGKVQIWDASRCKRTRTMEG-HRLRVG
A._thaliana_Cdh1.3                  NDSVCSVQWTREGSYISIGTS--HGQVQVWDGTQCKRVRTMGG-HQTRTG
V._carteri_Cdc20                    HDTVCSVEWSRRGTFLSVGTN--SGKVQIWDVAKLKLVRTLEG-HRARVG
C._hominis_Cdc20                    ---VTSVSWTQQGNHLAVGTR--QGSVQIWDVVEQKKVRTLNG-HRARIG
C._parvum_Cdc20                     ---VTSVSWTQQGNHLAVGTR--QGSVQIWDVVEQKKVRTLNG-HRARIG
C._muris_Cdc20                      QDLVTSVSWTQQGNHVAIGTR--QGSIQIWDVTVQKKVRTLGG-HRARVG
H._sapiens_Cdc20                    ---ISSVAWIKEGNYLAVGTS--SAEVQLWDVQQQKRLRNMTS-HSARVG
M._musculus_Cdc20                   ---ISSVAWIKEGNYLAVGTS--NAEVQLWDVQQQKRLRNMTS-HSARVS
D._rerio_Cdc20                      ---ICSVSWSKDGNFLAIGTS--DCKVELWDVQYQKRLRSMDG-HSARVG
```

```
D._melanogaster_fizzy                 ---AGSLSWIQEGQILAIGNS--TGAVELWDCSKVKRLRVMDG-HSARVG
A._thaliana_Cdc20.1                   ---VTSINWAPDGRHVAVGLN--NSEVQLWDSASNRQLRTLKGGHQSRVG
A._thaliana_Cdc20.2                   ---VTSINWAPDGRHVAVGLN--NSEVQLWDSASNRQLRTLKGGHQSRVG
A._thaliana_Cdc20.3                   ---VTSINWTQDGLDLAVGLD--NSEVQLWDFVSNRQVRTLIGGHESRVG
A._thaliana_Cdc20.4                   ---VTSINWTQDGLDLAVGLD--NSEVQLWDCVSNRQVRTLRGGHESRVG
A._thaliana_Cdc20.5                   ---VTSINWTQDGLDLAIGLD--NSEVQLWDCVSNRQVRTLRGGHESRVG
Micromonas_Cdc20                      --YVTSVQWGGDGKHIAVGTN--DAEVQIWDVSRLKQVRTLRG-HNARVG
P._yoelii_Cdc20                       ---ITSLKWNIFGNYLAVGLS--NGAVEIWDIEKGIKIRKYKN-HKLRVG
P._berghei_Cdc20                      -KNITSLKWNMFGNYLAVGLS--NGAVEIWDIEKGTKIRKYKN-HKLRVG
P._chaubaudi_Cdc20                    ---IASLKWNIFGNYLAVGLS--NGVVEIWDIEKGSKIRKYN--HKLRVG
P._falciparum_Cdc20                   ---ISSLKWNINGNFLATGLS--NGVVEIWDIEKCVRIRKYKN-HKSRVN
P._knowlesi_Cdc20                     --SITSLRWNFFGNHLSVGLS--NGVVQIWDLEKEVKIRKYRN-HKKRVG
P._vivax_Cdc20                        ---ITSLRWNLFGNHLAVGLS--NGAVQIWDLEKEVKIRKYRN-HKRRVG
S._cerevisiae_Ama1                    -EFFHSSNTNEKEPVFVFQTESFKGICCLEWFKPGEICKFYVGEENGNVS
                                           .         .            :                  .

S._cerevisiae_Cdc20                   SLSWL---DTLIATGSRSGEIQINDVRIKQH-----------------IV
S._pombe_Slp1                         CLSWN---RHVLSSGSRSGAIHHHDVRIANH-----------------QI
L._major_Cdc20                        SLSWEPLGGWLLASGSRDSTVVLRDVRERDTSTSASVASPSSSFSLASAT
L._infantum_Cdc20                     SLSWEPLGGWLLASGSRDSTVVLRDVRERDTSTSASAASPSSSSSLASAT
L._braziliensis_Cdc20                 SLSWDPLGGWLLASGSRDSTIVLRDVRERDT-TSASMSSASSFSSLASAT
T._brucei_Cdc20                       ALSWN---GSVLSSGSKDTTIRINDLR--DP----------------LGT
T._cruzi_Cdc20                        ALSWN---GSAIASGSKDASIRVNDLR--DP----------------VES
S._cerevisiae_Cdh1                    CLSWN---NHVLTSGSRDHRILHRDVRMPDP----------------FFE
S._pombe_Srw1                         ALSWN---DHVLSSGGRDNHILHRDVRAPEH----------------YFR
H._sapiens_Cdh1                       ALAWN---AEQLSSGSRDRMILQRDIRTPPL----------------QSE
M._musculus_Cdh1                      ALAWN---ADQLSSGSRDRMILQRDIRTPPL----------------QSE
D._rerio_fizzy-related                ALAWN---ADQLSSGSRDRMILQRDIRTPPL----------------QSE
D._melanogaster_fizzy-related         ALAWN---SDILSSGSRDRWIIQRDTRTPQL----------------QSE
C._elegans_fzr-1                      CLAWN---ADTICSGSRDRTIMHRDIRCDDN----------------DMG
C._briggsae_Cdh1                      CLAWN---ADTICSGSRDRTIIHRDIRAPDN----------------EEG
A._thaliana_Cdh1.1                    ALAWS---SSVLSSGSRDKSILQRDIRTQED----------------HVS
A._thaliana_Cdh1.2                    ALAWG---SSVLSSGSRDKSILQRDIRCQED----------------HVS
A._thaliana_Cdh1.3                    VLAWN---SRILSSGSRDRNILQHDIRVQSD----------------FVS
V._carteri_Cdc20                      TQAWG---SHVLCSGSRDRHILQRDIRCPEH----------------FTA
C._hominis_Cdc20                      AMDWC---GPILATGGRDHTVLLRDVREQEH----------------WCS
C._parvum_Cdc20                       AMDWC---GPILATGGRDHTVLLRDVREQEH----------------WCS
C._muris_Cdc20                        AMDWC---GPILATGGRDHTVLLRDVREQEH----------------WCN
H._sapiens_Cdc20                      SLSWN---SYILSSGSRSGHIHHHDVRVAEH-----------------HV
M._musculus_Cdc20                     SLSWN---SYILSSGSRSGHIHHHDVRVAEH-----------------HV
D._rerio_Cdc20                        CLSWN---DHILSSGSRSGLIHQHDVRVADH-----------------HI
D._melanogaster_fizzy                 SLAWN---SFLVSSGSRDGTIVHHDVRAREH-----------------KL
A._thaliana_Cdc20.1                   SLAWN---NHILTTGGMDGLIINNDVRIRSP-----------------IV
A._thaliana_Cdc20.2                   SLAWN---NHILTTGGMDGLIINNDVRIRSP-----------------IV
A._thaliana_Cdc20.3                   SLAWN---NHILTTGGMDGKIVNNDVRIRSS-----------------IV
A._thaliana_Cdc20.4                   SLAWD---NHILTTGGMDGKIVNNDVRIRSS-----------------IV
A._thaliana_Cdc20.5                   SLAWN---NHILTTGGMDGKIVNNDVRIRSS-----------------IV
Micromonas_Cdc20                      ALAWN---GTQLATGSRDNTVMMHDVRIREH-----------------RT
P._yoelii_Cdc20                       SLCWY---YNILTTGSRDNTIINCDVRTKDS-----------------NY
P._berghei_Cdc20                      ALCWY---YNILTTGSRDKTIINCDLRTKDS-----------------SY
P._chaubaudi_Cdc20                    SLCWY---YNILTTGSRDNTIINCDIRTKDS-----------------NY
P._falciparum_Cdc20                   TLCWN---HNTLTTGGRDNKIINSDIRSKEI-----------------YY
P._knowlesi_Cdc20                     ALGWY---YDTLTTGSKDNKIVCSDIRCKDS-----------------SY
P._vivax_Cdc20                        ALDWH---YNTLSTGSRDNKIVSLDIRCRES-----------------SY
S._cerevisiae_Ama1                    LFEIKS-LHFSIKNWSKRQKLEDENLIGLKL------------------H
                                                 : . .    :   :

S._cerevisiae_Cdc20                   STWAEHTGEVCGLSYKSDGLQLASGGNDNTVMIWDTRT------------
S._pombe_Slp1                         GTLQGHSSEVCGLAWRSDGLQLASGGNDNVVQIWDARS------------
L._major_Cdc20                        AVLRAHETEVCGLKWSPTGAMLASGGNDNQLLLWDRRSISTGSHSS-DTS
L._infantum_Cdc20                     AVLRAHETEVCGLKWSPTGAMLASGGNDNQLLLWDRRSISTGSRSS-DTS
L._braziliensis_Cdc20                 SVLRAHETEVCGLKWSPTGAMLASGGNDNQLLLWDRRSISTGSHSSGDTS
T._brucei_Cdc20                       WTLQAHRQSVCGLRWSPDGLRLASGGNDNQLLLWDMRTLSMN--------
T._cruzi_Cdc20                        WTLRCHQQSVCGLRWSPDGVRMASGGNDNQLLLWDSRTFSVR--------
S._cerevisiae_Cdh1                    -TIESHTQEVCGLKWNVADNKLASGGNDNVVHVYEGTS------------
S._pombe_Srw1                         -VLTAHRQEVCGLEWNSNENLLASGGNDNALMVWDKFE------------
H._sapiens_Cdh1                       RRLQGHRQEVCGLKWSTDHQLLASGGNDNKLLVWNHSS------------
M._musculus_Cdh1                      RRLQGHRQEVCGLKWSTDHQLLASGGNDNKLLVWNHSS------------
D._rerio_fizzy-related                RRLQGHRQEVCGLKWSTDHQLLASGGNDNKLLVWNHSS------------
```

```
D._melanogaster_fizzy-related      RRLAGHRQEVCGLKWSPDNQYLASGGNDNRLYVWNQHS------------
C._elegans_fzr-1                   RKLTNHRQEVCGLKWSPDKQLLASGGNDNQLLVWNLRR------------
C._briggsae_Cdh1                   RKMTHHRQEVCGLKWSPDKQLLASGGNDNQLLVWNLRR------------
A._thaliana_Cdh1.1                 -KLKGHKSEICGLKWSSDNRELASGGNDNKLFVWNQHS------------
A._thaliana_Cdh1.2                 -KLAGHKSEVCGLKWSYDNRELASGGNDNRLFVWNQHS------------
A._thaliana_Cdh1.3                 -KLVGHKSEVCGLKWSHDDRELASGGNDNQLLVWNNHS------------
V._carteri_Cdc20                   -KLVGHRSEVCGLKWSPDDRQLASGGNDNQLYIWSLPS------------
C._hominis_Cdc20                   -RWLGHKQEVCGVKWSPNEMQLATGGNDNKLLIWSQGY------------
C._parvum_Cdc20                    -RWLGHKQEVCGVKWSPNEMQLATGGNDNKLLIWSQGY------------
C._muris_Cdc20                     -RWLGHKQEVCGVKWSPNEMQLATGGNDNKLLIWSQGY------------
H._sapiens_Cdc20                   ATLSGHSQEVCGLRWAPDGRHLASGGNDNLVNVWP-SAPGEGGWV-----
M._musculus_Cdc20                  ATLSGHSQEVCGLRWAPDGRHLASGGNDNIVNVWP-SGPGESGWA-----
D._rerio_Cdc20                     FTFGGHTQEVCGLTWSPDGRYLASGGNDNMMYIWP-MTTG-SENQ-----
D._melanogaster_fizzy              STLSGHTQEVCGLKWSTDFKYLASGGNDNLVNVWS-AASGGVGTAT----
A._thaliana_Cdc20.1                ETYRGHTQEVCGLKWSGSGQQLASGGNDNVVHIWD-RSVASSNSTT----
A._thaliana_Cdc20.2                ETYRGHTQEVCGLKWSGSGQQLASGGNDNVVHIWD-RSVASSNSTT----
A._thaliana_Cdc20.3                GTYLGHTEEVCGLKWSESGKKLASGGNYNVVHIWDHRSVASSKPTR----
A._thaliana_Cdc20.4                ETYLGHTEEVCGLKWSESGNKQASGGNDNVVHIWD-RSLASSKQTR----
A._thaliana_Cdc20.5                ETYLGHTEEVCGLKWSESGKKLASGGNDNVVHIWDHRSVASSNPTR----
Micromonas_Cdc20                   ATLTSHSQEVCGLKWAPSGNQLASGGNDNLLHIWDQNSIGNG--------
P._yoelii_Cdc20                    IKYEKHTSEVCGLQWNYNGKLLASGSNDNSIYLWDHNK------------
P._berghei_Cdc20                   IKYEKHTSEVCGLQWNYNGKLLASGSNDNSIYLWDNNK------------
P._chaubaudi_Cdc20                 IKYEKHTSEVCGLQWNYNGKLLASGSNDNSIYIWDNNK------------
P._falciparum_Cdc20                IELTKHKSEICGLEWNADGTYLASGSNDNSIYIWDKYT------------
P._knowlesi_Cdc20                  AQLTNHTSEVCGLQWNYQTKQLASGSNDNSVYIWEWRK------------
P._vivax_Cdc20                     AQLSNHSSEVCGLLWNYKTKQLASGSNDNSVCIWEERK------------
S._cerevisiae_Ama1                 STYQAQAQQVCGISLNEHANLLAVGGNDNSCSLWDISD------------
                                        :  .:**:         * *.* *   ::

S._cerevisiae_Cdc20                -------SLPQFSKKT-HTAAVKALSWCPYSPNILASGGGQTDKHIHFWN
S._pombe_Slp1                      -------SIPKFTKTN-HNAAVKAVAWCPWQSNLLATGGGTMDKQIHFWN
L._major_Cdc20                     GAYRHGECQPIFFLNK-HTAAVKALSWNPTQPALLASGGGSHDKALRFWN
L._infantum_Cdc20                  GVYRHGECQPIFFLNK-HTAAVKALSWNPAQPALLASGGGSHDKALRFWN
L._braziliensis_Cdc20              GIHRHGECRPIFFLNK-HTAAVKALSWNPTQPALLASGGGSHDKALRFWN
T._brucei_Cdc20                    -------STPSMLLNK-HTAAVKAIAWNPVQHNLLVSGGGSDDKMLRFWN
T._cruzi_Cdc20                     -------SQPVLRLNK-HTAAVKAIAWNPVQHNLLLSGGGSEDKMLRFWN
S._cerevisiae_Cdh1                 -------KSPILTFDE-HKAAVKAMAWSPHKRGVLATGGGTADRRLKIWN
S._pombe_Srw1                      -------EKPLYSFHN-HIAAVKAITWSPHQRGILASGGGTADRTIKLWN
H._sapiens_Cdh1                    -------LSPVQQYTE-HLAAVKAIAWSPHQHGLLASGGGTADRCIRFWN
M._musculus_Cdh1                   -------LSPVQQYTE-HLAAVKAIAWSPHQHGLLASGGGTADRCIRFWN
D._rerio_fizzy-related             -------VLPMQQYTE-HLAAVKAIAWSPHQHGLLASGGGTADRCIRFWN
D._melanogaster_fizzy-related      -------VNPVQSYTE-HMAAVKAIAWSPHHHGLLASGGGTADRCIRFWN
C._elegans_fzr-1                   -------NEPIQTYTQ-HNAAVKALAWSPHHHGLLVSGGGTADRCLRFWN
C._briggsae_Cdh1                   -------PDPLQTYTQ-HNAAVKALAWSPHHHGLLVSGGGTADRCLRFWN
A._thaliana_Cdh1.1                 -------TQPVLRFCE-HAAAVKAIAWSPHHFGLLASGGGTADRCIRFWN
A._thaliana_Cdh1.2                 -------TQPVLKYSE-HTAAVKAIAWSPHVHGLLASGGGTADRCIRFWN
A._thaliana_Cdh1.3                 -------QQPILKLTE-HTAAVKAITWSPHQSSLLASGGGTADRCIRFWN
V._carteri_Cdc20                   -------SSPVYKFAD-HTAAVKAIAWSPHQHSLLASGGGTADRCIRFWN
C._hominis_Cdc20                   -------DTPVCQFQE-HNAAVKALSWNPHQSGLLASGGGTADRHIRIWN
C._parvum_Cdc20                    -------DTPVCQFQE-HTAAVKALSWNPHQSGLLASGGGTADRHIRIWN
C._muris_Cdc20                     -------ETPVCQFQE-HTAAVKALSWSPHQSGLLASGGGTADRHIRVWN
H._sapiens_Cdc20                   ---------PLQTFTQ-HQGAVKAVAWCPWQSNVLATGGGTSDRHIRIWN
M._musculus_Cdc20                  ---------PLQTFTQ-HQGAVKAVAWCPWQSNILATGGGTSDRHIRIWN
D._rerio_Cdc20                     ---------AIHALSE-HQGAVKALAWCPWQPNIPASGGGTSDRHIRIWN
D._melanogaster_fizzy              --------DPLHKFND-HQAAVRALAWCPWQPSTLASGGGTADRCIKFWN
A._thaliana_Cdc20.1                --------QWLHRLEE-HTSAVKALAWCPFQANLLATGGGGGDRTIKFWN
A._thaliana_Cdc20.2                --------QWLHRLEE-HTSAVKALAWCPFQANLLATGGGGGDRTIKFWN
A._thaliana_Cdc20.3                --------QWLHRFEE-HTAAVRALAWCPFQATLLATGGGVGDGKIKFWN
A._thaliana_Cdc20.4                --------QWLHRFEE-HTAAVRALAWCPFQASLLATGGGVGDGKIKFWN
A._thaliana_Cdc20.5                --------QWLHRFEE-HTAAVRALAWCPFQASLLATGGGVGDGKIKFWN
Micromonas_Cdc20                   --------THLHRLDA-HQAAVKALAWCPFQSNLLASGGGTADRCIKFWN
P._yoelii_Cdc20                    -------NNSIFHFTK-HKAAVKAISWCPHDHNLLTTGGGSADKKIYFWN
P._berghei_Cdc20                   -------NNSIFHFTK-HKAAVKAISWCPHDHNLLTTGGGSTDKKIYFWN
P._chaubaudi_Cdc20                 -------NDFIFHFTK-HKAAVKAISWCPHDHNLLTTGGGSADKKIYFWD
P._falciparum_Cdc20                -------NKYLFHFKK-HKAAVKAIAWCPYKNHILSSGGGSVDKKIFLWN
P._knowlesi_Cdc20                  -------CVPLFQLTK-HTAAVKAMSWSPHKENLLATGGGSADKKIFLWN
P._vivax_Cdc20                     -------WAPLFQFTK-HTAAVKAMSWSPHQHNLLATGGGSADKHIFFWD
S._cerevisiae_Ama1                 ------LDKPIKKFVLPHKAAVKAIAFCPWSKSLLATGGGSKDRCIKFWH
                                                    * .**:*::: *       :***  *  : .*.
```

```
S._cerevisiae_Cdc20                SITGARVGSINTGSQVSSLHWGQSHTSTNGGMMNKEIVATGGNPENA---
S._pombe_Slp1                      AATGARVNTVDAGSQVTSLIWSP-HS---------KEIMSTHGFPDNN---
L._major_Cdc20                     SLTGECVHHINTGSQVCGVVWNRAGT----------ELVTAHGYTDNQ---
L._infantum_Cdc20                  SLTGECVHHINTGSQVCGVVWNRVGT----------ELVTAHGYTDNQ---
L._braziliensis_Cdc20              SLTGECVHHINTGSQVCGVVWSRTGT----------ELVTAHGYTDNQ---
T._brucei_Cdc20                    TSTGECISNFNAESQVCGVLWNHGGT----------ELVSSHGYSHNR---
T._cruzi_Cdc20                     TSTGECINCHNAESQVCGVLWNLSGT----------ELVSSHGFSHNR---
S._cerevisiae_Cdh1                 VNTSIKMSDIDSGSQICNMVWSKNTN----------ELVTSHGYSKYN---
S._pombe_Srw1                      TQRGSMLHNIDTGSQVCNLLWSKQTN----------EFISTHGFMENE---
H._sapiens_Cdh1                    TLTGQPLQCIDTGSQVCNLAWSKHAN----------ELVSTHGYSQNQ---
M._musculus_Cdh1                   TLTGQPLQCIDTGSQVCNLAWSKHAN----------ELVSTHGYSQNQ---
D._rerio_fizzy-related             TLTAQPLQCIDTGSQVCNLAWSKHTN----------ELVSTHGYSQNQ---
D._melanogaster_fizzy-related      TLTGQPMQCVDTGSQVCNLAWSKHSS----------ELVSTHGYSQNQ---
C._elegans_fzr-1                   TLTAQPMQCVDTGSQVCNVAWSKHSS----------ELVSTHGYSFNH---
C._briggsae_Cdh1                   TLTAQPMQCVDTGSQVCNVAWSKHSS----------ELVSTHGYSYNH---
A._thaliana_Cdh1.1                 TTTNTHLNCVDTNSQVCNLVWSKNVN----------ELVSTHGYSQNQ---
A._thaliana_Cdh1.2                 TTTNTHLSSIDTCSQVCNLAWSKNVN----------ELVSTHGYSQNQ---
A._thaliana_Cdh1.3                 TTNGNQLNSIDTGSQVCNLAWSKNVN----------EIVSTHGYSQNQ---
V._carteri_Cdc20                   TATGMPLNCIDTGSQVCNISWSKNAN----------EIVSTHGYSQNQ---
C._hominis_Cdc20                   TVTNSCVMAVDTGSQVCNIAWSGNVN----------ELVSTHGYSLNQ---
C._parvum_Cdc20                    TVTNSCVMAVDTGSQVCNIAWSGNVN----------ELVSTHGYSLNQ---
C._muris_Cdc20                     TVTNCCVMAVDTGSQVCNIAWSGNVN----------ELVSTHGYSLNQ---
H._sapiens_Cdc20                   VCSGACLSAVDAHSQVCSILWSPHYK----------ELISGHGFAQNQ---
M._musculus_Cdc20                  VCSGACLSAVDVHSQVCSILWSPHYK----------ELISGHGFAQNQ---
D._rerio_Cdc20                     ASSGSCISSLDTCSQVSSLVFAPNYK----------ELVSGHGFAHDK---
D._melanogaster_fizzy              VNNGTLMKSVDSKSQVCSLLFSRHYK----------ELISAHGFANNQ---
A._thaliana_Cdc20.1                THTGACLNSVDTGSQVCSLLWSKNER----------ELLSSHGFTQNQ---
A._thaliana_Cdc20.2                THTGACLNSVDTGSQVCSLLWSKNER----------ELLSSHGFTQNQ---
A._thaliana_Cdc20.3                THTGACLNSVETGSQVCSLLWSQRER----------ELLSSHGFTQNQ---
A._thaliana_Cdc20.4                THTGACLNSVETGSQVCSLLWSQSER----------ELLSSHGFTQNQ---
A._thaliana_Cdc20.5                THTGACLNSVETGSQVCSLLWSKSER----------ELLSSHGFTQNQ---
Micromonas_Cdc20                   TNTGALLNSIDTHSQVCSLQWNKHER----------ELLSSHGYSQNQ---
P._yoelii_Cdc20                    VNNGECINSINTNSQVSNILWSKNTK----------EFISTHSYTHSQ---
P._berghei_Cdc20                   INNGECINSINTNSQVSNILWSKNTK----------EFISTHSYTHSQ---
P._chaubaudi_Cdc20                 INNGECINSINTKSQVSNILWSKNTK----------ELISTHSYTHSQ---
P._falciparum_Cdc20                IKTGKSINEIYTKSQVSNIIWSINTS----------ELISTHSHSLNQ---
P._knowlesi_Cdc20                  TSTGKCLDEVRANSQVSNIFWSKHTE----------EFVSTHSYSLGQ---
P._vivax_Cdc20                     TSTGECLNELATSSQVSNLFWSKHSE----------ELVSTHSYSLGQ---
S._cerevisiae_Ama1                 TSTGTLLDEIYTSGQVTSLIWSLRHK----------QIVATFGFGDTKNPV
                                         :      .*: .: :              ::::  .

S._cerevisiae_Cdc20                -ISVYNYETKFKVAEV-VHAHEARICCSQLSPDGTTLATVGGDENLKFYK
S._pombe_Slp1                      -LSIWSYSSSGLTKQVDIPAHDTRVLYSALSPDGRILSTAASDENLKFWR
L._major_Cdc20                     -LSIWRYPSLRRIANL--IGHTSRVLHLALSADGETVVSAAGDETLRFWR
L._infantum_Cdc20                  -LSIWRYPSLRRIANL--IGHTSRVLHLALSADGQTVVSAAGDETLRFWR
L._braziliensis_Cdc20              -LSIWRYPSLRRIANL--IGHTSRVLHLALSADGQTVVSAAGDETLRFWR
T._brucei_Cdc20                    -LTIWKYPTMRRVADL--AGHTSRVLHMCMSTDGEVVVSAAADETIRFWR
T._cruzi_Cdc20                     -LTIWKYPTMRRVADL--TGHTSRVLHLCMSTDGEVVASAAGDETIRFWR
S._cerevisiae_Cdh1                 -LTLWDCNSMDPIAIL--KGHSFRVLHLTLSNDGTTVVSGAGDETLRYWK
S._pombe_Srw1                      -VALWNYPSVSRVGTL--KGHTDRVLYLAMSPNGENIVTGAADETLRFWK
H._sapiens_Cdh1                    -ILVWKYPSLTQVAKL--TGHSYRVLYLAMSPDGEAIVTGAGDETLRFWN
M._musculus_Cdh1                   -ILVWKYPSLTQVAKL--TGHSYRVLYLAMSPDGEAIVTGAGDETLRFWN
D._rerio_fizzy-related             -ILVWKYPSLTQVAKL--TGHSYRVLYLAMSPDGEAIVTGAGDETLRFWN
D._melanogaster_fizzy-related      -ILVWKYPSLTQVAKL--TGHSYRVLYLALSPDGEAIVTGAGDETLRFWN
C._elegans_fzr-1                   -VIIWKYPSLQPVTKL--VGHQYRVLYLAMSPDGESIVTGAGDETLRFW-
C._briggsae_Cdh1                   -VIIWKYPSLQPVTKL--VGHQFRVLYLAMSPDGESIVTGAGDETLRFW-
A._thaliana_Cdh1.1                 -IIVWKYPTMSKLATL--TGHSYRVLYLAVSPDGQTIVTGAGDETLRFWN
A._thaliana_Cdh1.2                 -IIVWKYPTMSKIATL--TGHTYRVLYLAVSPDGQTIVTGAGDETLRFWN
A._thaliana_Cdh1.3                 -IMLWKYPSMSKVATL--TGHSMRVLYLATSPDGQTIVTGAGDETLRFWN
V._carteri_Cdc20                   -VIIWKYPSMAKLATL--TGHTLRVLYLAVSPDGQTIVTGAGDETLRFWS
C._hominis_Cdc20                   -VILWKWPSMQKIATL--TGHTYRVLYLAVSPDGQTIVTGAGDETLRFWQ
C._parvum_Cdc20                    -VILWKWPSMQKIATL--TGHTYRVLYLAVSPDGQTIVTGAGDETLRFWQ
C._muris_Cdc20                     -VILWKWPSMQKIVTL--TGHTYRVLYLAVSPDGQTIVTGAGDETLRFWQ
H._sapiens_Cdc20                   -LVIWKYPTMAKVAEL--KGHTSRVLSLTMSPDGATVASAAADETLRLWR
M._musculus_Cdc20                  -LVIWKYPTMAKVAEL--KGHTARVLGLTMSPDGATVASAAADETLRLWR
D._rerio_Cdc20                     -VVIWKYPSFAKVTEH--EGHEARILNLALSPDGSTLASIAADETIRLWK
D._melanogaster_fizzy              -LTIWKYPTMVKQADL--TGHTSRVLQMAMSPDGSTVISAGADETLRLWN
A._thaliana_Cdc20.1                -LTLWKYPSMVKMAEL--TGHTSRVLYMAQSPDGCTVASAAGDETLRFWN
```

```
A._thaliana_Cdc20.2                -LTLWKYPSMVKMAEL--TGHTSRVLYMAQSPDGCTVASAAGDETL----
A._thaliana_Cdc20.3                -LTLWKYPSMSKMAEL--NGHTSRVLFMAQSPNGCTVASAAGDENLRLWN
A._thaliana_Cdc20.4                -LTLWKYPSMSKMAEL--NGHTSRVLFMAQSPNGCTVASAAGDENLRLWN
A._thaliana_Cdc20.5                -LTLWKYPSMVKMAEL--NGHTSRVLFMAQSPDGCTVASAAGDETLRLWN
Micromonas_Cdc20                   -LCLWKYPTMTKMAEL--TGHSARVLHMAQSPDGTTVVSAAADETLRFWK
P._yoelii_Cdc20                    -IIIWNYPNLNKISAL--TDHKLRVLYAALSPDGTSLVSGSPDETIRLWN
P._berghei_Cdc20                   -IIIWNYPDLNKISAL--TDHKLRVLYAALSPDGTSLVSGSPDETIRLWN
P._chaubaudi_Cdc20                 -IIIWNYPDLNKISAL--TDHKLRVLYAALSPDGTSLVSGSPDETIRLWN
P._falciparum_Cdc20                -IILWNLPQLKKVTTL--RGHKSRVLYAALSPDGTSIATGSPDQTIRLWN
P._knowlesi_Cdc20                  -VVLWKYPRLKKVSAL--SGHALRVLYGALSPDGESIVTGSPDETLRLWR
P._vivax_Cdc20                     -VVLWKYPRLQKVSTL--SGHALRVLYGALSPDGESLVTGSPDETLRLWR
S._cerevisiae_Ama1                 LITLYSYPKLSKLLEVR-SPNPLRVLSAVISPSSMAICVATNDETIRFYE
                                    : ::               :  *:     * ..  :     *:.:

S._cerevisiae_Cdc20                ------
S._pombe_Slp1                      ------
L._major_Cdc20                     ------
L._infantum_Cdc20                  ------
L._braziliensis_Cdc20              ------
T._brucei_Cdc20                    ------
T._cruzi_Cdc20                     ------
S._cerevisiae_Cdh1                 ------
S._pombe_Srw1                      ------
H._sapiens_Cdh1                    ------
M._musculus_Cdh1                   ------
D._rerio_fizzy-related             ------
D._melanogaster_fizzy-related      ------
C._elegans_fzr-1                   ------
C._briggsae_Cdh1                   ------
A._thaliana_Cdh1.1                 ------
A._thaliana_Cdh1.2                 ------
A._thaliana_Cdh1.3                 ------
V._carteri_Cdc20                   ------
C._hominis_Cdc20                   I-----
C._parvum_Cdc20                    I-----
C._muris_Cdc20                     ------
H._sapiens_Cdc20                   ------
M._musculus_Cdc20                  ------
D._rerio_Cdc20                     ------
D._melanogaster_fizzy              ------
A._thaliana_Cdc20.1                ------
A._thaliana_Cdc20.2                ------
A._thaliana_Cdc20.3                ------
A._thaliana_Cdc20.4                ------
A._thaliana_Cdc20.5                ------
Micromonas_Cdc20                   ------
P._yoelii_Cdc20                    ------
P._berghei_Cdc20                   V-----
P._chaubaudi_Cdc20                 ------
P._falciparum_Cdc20                I-----
P._knowlesi_Cdc20                  ------
P._vivax_Cdc20                     ------
S._cerevisiae_Ama1                 LWNDKE
```
