# Supplementary material for: A Putative Homologue of CDC20/CDH1 in the Malaria Parasite Is Essential for Male Gamete Development
Source: PLoS Pathog. 2012 Feb 23;8(2):e1002554. doi: 10.1371/journal.ppat.1002554 (PMC3285604; doi:10.1371/journal.ppat.1002554)
Supplement: Figure S2 — gfp tagging and targeted disruption of the Pbcdc20 locus. A. Schematic representation of the gene targeting strategy used for gene tagging the endogenous locus with gfp via single homologous recombination. Primers 1+2 used for diagnostic PCR are indicated, as well as the EcoRI site used for Southern blotting. Probe location used for detection by Southern blotting is indicated. B. Diagnostic PCR confirming successful integration of the tagging sequence. C. Southern blot analysis of EcoRI digested T36 genomic DNA using the 3′ UTR of the targeting construct as a probe. Band sizes for CDC20-GFP (tag) and wild-type (wt) are indicated. D. Western blot analysis using an anti-GFP antibody against control wild-type-GFP (wt) and transgenic (tag) activated gametocytes showing bands of expected sizes of 29 kDa for wild-type-GFP and 92 kDa for PbCDC20-GFP. E. Schematic representation of the gene targeting strategy used for gene disruption via double homologous recombination. Primers 1–4 used for diagnostic PCR are indicated, as well as the HindIII digestion site used for Southern blotting. Probe location used for detection by Southern blotting is indicated. F. Diagnostic PCR confirming successful integration of the disruption sequence of cdc20 in mutants N10 clone 7 (cl7) and N10 clone 9 (cl9). Primers 1+2 were used to verify successful integration at the correct locus. Primers 3+4 were used to confirm loss of the endogenous gene. G. Southern blot analysis of HindIII digested N10 clone 7 genomic DNA using the 5′ UTR of the targeting construct as a probe. Band sizes for N10 clone 7 (cl7) and wild-type (wt) are indicated. H. Pulse-field gel electrophoresis blot hybridised with Pb 3′UTR which detects the endogenous chromosome 7 locus and disrupted locus on chromosome 5 in both clones. I. Bar graph showing relative expression of endogenous Pbcdc20 in Δcdc20 mutants using qRT-PCR compared to wild-type. Error bars represent ±SEM, n = 3 from three separate experiments in both clone 7 a [file ppat.1002554.s002.doc]

**CLUSTAL 2.1 multiple sequence alignment**

S._cerevisiae_Cdc20 ---SKK-NVLAIALDTALY-LWNATTGDVSLLTDFENTT-----------

S._pombe_Slp1 LDWSNL-NVVAVALERNVY-VWNADSGSVSALAETDESTY----------

L._major_Cdc20 IDWSATSDVLCVALQNCVY-LWDAKTCGITELPRVVPTGGGLHGDGRSGD

L._infantum_Cdc20 IDWSATSDVLCVALQNCVY-LWDAKTCGITELPRVVSTGGGLHGDGRSGD

L._braziliensis_Cdc20 IDWSATSDILGVALQNCVY-LWNAKTCDITELPRVVSTGSGMHGEGRSAN

T._brucei_Cdc20 MDWSAK-DVLAVGLQGSVY-LWYEKTSNIAQLPCQRPANG----------

T._cruzi_Cdc20 IDWSSK-DILAVGLQGAVY-LWDAKTCNITHLPCQRPPNG----------

S._cerevisiae_Cdh1 IDWSST-DVLAVALGKSIF-LTDNNTGDVVHLCDTEN-------------

S._pombe_Srw1 -----------------------------------TD-------------

H._sapiens_Cdh1 VDWSSL-NVLSVGLGTCVY-LWSACTSQVTRLCDLSV--E----------

M._musculus_Cdh1 VDWSSL-NVLSVGLGTCVY-LWSACTSQVTRLCDLSV--E----------

D._rerio_fizzy-related VDWSSL-NVLSVGLGTCVY-LWSACTSQVTRLCDLSV--E----------

D._melanogaster_fizzy-related VDWSSQ-NVLAVGLGSCVY-LWSACTSQVTRLCDLSP--D----------

C._elegans_fzr-1 VDWSSQ-NQLSVGLAACVY-LWSATTSQVIKLCDLGQTNE----------

C._briggsae_Cdh1 VDWSSQ-NQLSVGLSTCVY-LWSATTSQVIKLCDLSASNE----------

A._thaliana_Cdh1.1 VDWSAQ-NVLAVGLGNCVY-LWNACSSKVTKLCDLGV-------------

A._thaliana_Cdh1.2 VDWSAQ-NVLAVGLGNCVY-LWNACSSKVTKLCDLGA-------------

A._thaliana_Cdh1.3 ------------------Y-LWTASNSKVTKLCDLGP-------------

V._carteri_Cdc20 VDWSSQ-NVLAVGLGTCVY-LWSAMSSTVTKLCDLAP-------------

C._hominis_Cdc20 --------------------------------------------------

C._parvum_Cdc20 --------------------------------------------------

C._muris_Cdc20 VDWSST-NLLAVGLSSSLY-LWNASTSKVTNLMSLPE-------------

H._sapiens_Cdc20 VDWSSG-NVLAVALDNSVY-LWSASSGDILQLLQMEQPGEY---------

M._musculus_Cdc20 VDWSSG-NVLAVALDNSVY-LWNAGSGDILQLLQMEQPGDY---------

D._rerio_Cdc20 -----Q-NVLAVGLANQVY-LWDAGEGDIVLLKKMEDDNEY---------

D._melanogaster_fizzy MDWSAD-NIVAVALGSCVY-LWNAQTGNIEQLTEFEE-GDY---------

A._thaliana_Cdc20.1 --------------DHTVY-LWDASTGSTSELVTIDEEKGP---------

A._thaliana_Cdc20.2 --------------DHTVY-LWDASTGSTSELVTIDEEKGP---------

A._thaliana_Cdc20.3 -------NVLAIALGDTVY-LWDASSGSTSELVTIDEDKGP---------

A._thaliana_Cdc20.4 -------NVLAIALGDTVY-LWDASSGSTSELVTIDEDKGP---------

A._thaliana_Cdc20.5 ----SS-NVLAIALGDTVY-LWDASSGSTYKLVTIDEEEGP---------

Micromonas_Cdc20 -----------------VY-LWNADSGDIQQLCQTDPNNGDD--------

P._yoelii_Cdc20 --------------------------------------------------

P._berghei_Cdc20 --------------------------------------------------

P._chaubaudi_Cdc20 --------------------------------------------------

P._falciparum_Cdc20 --------------------------------------------------

P._knowlesi_Cdc20 --------------------------------------------------

P._vivax_Cdc20 --------------------------------------------------

S._cerevisiae_Ama1 HQYLSEKRDLVTCVSFCPYNTYFIVGTKFGRILLYDQK------------

S._cerevisiae_Cdc20 ---ICSVTWSDDDCHISIGKE--DGNTEIWDVETMSLIRTMRSGLGVRIG

S._pombe_Slp1 ---VASVKWSHDGSFLSVGLG--NGLVDIYDVESQTKLRTM-AGHQARVG

L._major_Cdc20 AQLVCGLNWAPDGCHLAVGGH--SGAVEVWDVETQQIVHTYRQ-HADRTV

L._infantum_Cdc20 AQLVCGLNWAPDGCHLAVGRH--SGAVEVWDVETQQIVHTYRQ-HADRTV

L._braziliensis_Cdc20 AQLVCGLNWAPDGRHLAIGRN--SGAVEVWDVEAQRIVHTYRQ-HADRTV

T._brucei_Cdc20 --IICGVSWSEDGNHLALGAD--DGSVEIWDVEAERITRRLHH-HTDRVG

T._cruzi_Cdc20 --IFCGVTWSEDGNLLALGTD--DGSLEIWDVEMQRITRRLYQ-HTDRVG

S._cerevisiae_Cdh1 --EYTSLSWIGAGSHLAVGQA--NGLVEIYDVMKRKCIRTLSG-HIDRVA

S._pombe_Srw1 --TVTSLRWVQRGTHLAVGTH--NGSVEIWDAATCKKTRTMSG-HTERVG

H._sapiens_Cdh1 GDSVTSVGWSERGNLVAVGTH--KGFVQIWDAAAGKKLSMLEG-HTARVG

M._musculus_Cdh1 GDSVTSVGWSERGNLVAVGTH--KGFVQIWDAAAGKKLSMLEG-HTARVG

D._rerio_fizzy-related GDSVTSVGWSERGNLVAVGTH--KGFVQIWDATAGKKLFALEG-HTARVG

D._melanogaster_fizzy-related ANTVTSVSWNERGNTVAVGTH--HGYVTVWDVAANKQINKLNG-HSARVG

C._elegans_fzr-1 QDQVTSVQWCDKGDLLAVGTS--RGVTQIWDVTTQKKTRELTG-HSSRVG

C._briggsae_Cdh1 QDQVTSVQWCDKGDLLAVGTN--RGITQIWDVTTQKKIRDLGG-HTSRVG

A._thaliana_Cdh1.1 DETVCSVGWALRGTHLAIGTS--SGTVQIWDVLRCKNIRTMEG-HRLRVG

A._thaliana_Cdh1.2 EDSVCSVGWALRGTHLAVGTS--TGKVQIWDASRCKRTRTMEG-HRLRVG

A._thaliana_Cdh1.3 NDSVCSVQWTREGSYISIGTS--HGQVQVWDGTQCKRVRTMGG-HQTRTG

V._carteri_Cdc20 HDTVCSVEWSRRGTFLSVGTN--SGKVQIWDVAKLKLVRTLEG-HRARVG

C._hominis_Cdc20 ---VTSVSWTQQGNHLAVGTR--QGSVQIWDVVEQKKVRTLNG-HRARIG

C._parvum_Cdc20 ---VTSVSWTQQGNHLAVGTR--QGSVQIWDVVEQKKVRTLNG-HRARIG

C._muris_Cdc20 QDLVTSVSWTQQGNHVAIGTR--QGSIQIWDVTVQKKVRTLGG-HRARVG

H._sapiens_Cdc20 ---ISSVAWIKEGNYLAVGTS--SAEVQLWDVQQQKRLRNMTS-HSARVG

M._musculus_Cdc20 ---ISSVAWIKEGNYLAVGTS--NAEVQLWDVQQQKRLRNMTS-HSARVS

D._rerio_Cdc20 ---ICSVSWSKDGNFLAIGTS--DCKVELWDVQYQKRLRSMDG-HSARVG

D._melanogaster_fizzy ---AGSLSWIQEGQILAIGNS--TGAVELWDCSKVKRLRVMDG-HSARVG

A._thaliana_Cdc20.1 ---VTSINWAPDGRHVAVGLN--NSEVQLWDSASNRQLRTLKGGHQSRVG

A._thaliana_Cdc20.2 ---VTSINWAPDGRHVAVGLN--NSEVQLWDSASNRQLRTLKGGHQSRVG

A._thaliana_Cdc20.3 ---VTSINWTQDGLDLAVGLD--NSEVQLWDFVSNRQVRTLIGGHESRVG

A._thaliana_Cdc20.4 ---VTSINWTQDGLDLAVGLD--NSEVQLWDCVSNRQVRTLRGGHESRVG

A._thaliana_Cdc20.5 ---VTSINWTQDGLDLAIGLD--NSEVQLWDCVSNRQVRTLRGGHESRVG

Micromonas_Cdc20 --YVTSVQWGGDGKHIAVGTN--DAEVQIWDVSRLKQVRTLRG-HNARVG

P._yoelii_Cdc20 ---ITSLKWNIFGNYLAVGLS--NGAVEIWDIEKGIKIRKYKN-HKLRVG

P._berghei_Cdc20 -KNITSLKWNMFGNYLAVGLS--NGAVEIWDIEKGTKIRKYKN-HKLRVG

P._chaubaudi_Cdc20 ---IASLKWNIFGNYLAVGLS--NGVVEIWDIEKGSKIRKYN--HKLRVG

P._falciparum_Cdc20 ---ISSLKWNINGNFLATGLS--NGVVEIWDIEKCVRIRKYKN-HKSRVN

P._knowlesi_Cdc20 --SITSLRWNFFGNHLSVGLS--NGVVQIWDLEKEVKIRKYRN-HKKRVG

P._vivax_Cdc20 ---ITSLRWNLFGNHLAVGLS--NGAVQIWDLEKEVKIRKYRN-HKRRVG

S._cerevisiae_Ama1 -EFFHSSNTNEKEPVFVFQTESFKGICCLEWFKPGEICKFYVGEENGNVS

. . : .

S._cerevisiae_Cdc20 SLSWL---DTLIATGSRSGEIQINDVRIKQH-----------------IV

S._pombe_Slp1 CLSWN---RHVLSSGSRSGAIHHHDVRIANH-----------------QI

L._major_Cdc20 SLSWEPLGGWLLASGSRDSTVVLRDVRERDTSTSASVASPSSSFSLASAT

L._infantum_Cdc20 SLSWEPLGGWLLASGSRDSTVVLRDVRERDTSTSASAASPSSSSSLASAT

L._braziliensis_Cdc20 SLSWDPLGGWLLASGSRDSTIVLRDVRERDT-TSASMSSASSFSSLASAT

T._brucei_Cdc20 ALSWN---GSVLSSGSKDTTIRINDLR--DP----------------LGT

T._cruzi_Cdc20 ALSWN---GSAIASGSKDASIRVNDLR--DP----------------VES

S._cerevisiae_Cdh1 CLSWN---NHVLTSGSRDHRILHRDVRMPDP----------------FFE

S._pombe_Srw1 ALSWN---DHVLSSGGRDNHILHRDVRAPEH----------------YFR

H._sapiens_Cdh1 ALAWN---AEQLSSGSRDRMILQRDIRTPPL----------------QSE

M._musculus_Cdh1 ALAWN---ADQLSSGSRDRMILQRDIRTPPL----------------QSE

D._rerio_fizzy-related ALAWN---ADQLSSGSRDRMILQRDIRTPPL----------------QSE

D._melanogaster_fizzy-related ALAWN---SDILSSGSRDRWIIQRDTRTPQL----------------QSE

C._elegans_fzr-1 CLAWN---ADTICSGSRDRTIMHRDIRCDDN----------------DMG

C._briggsae_Cdh1 CLAWN---ADTICSGSRDRTIIHRDIRAPDN----------------EEG

A._thaliana_Cdh1.1 ALAWS---SSVLSSGSRDKSILQRDIRTQED----------------HVS

A._thaliana_Cdh1.2 ALAWG---SSVLSSGSRDKSILQRDIRCQED----------------HVS

A._thaliana_Cdh1.3 VLAWN---SRILSSGSRDRNILQHDIRVQSD----------------FVS

V._carteri_Cdc20 TQAWG---SHVLCSGSRDRHILQRDIRCPEH----------------FTA

C._hominis_Cdc20 AMDWC---GPILATGGRDHTVLLRDVREQEH----------------WCS

C._parvum_Cdc20 AMDWC---GPILATGGRDHTVLLRDVREQEH----------------WCS

C._muris_Cdc20 AMDWC---GPILATGGRDHTVLLRDVREQEH----------------WCN

H._sapiens_Cdc20 SLSWN---SYILSSGSRSGHIHHHDVRVAEH-----------------HV

M._musculus_Cdc20 SLSWN---SYILSSGSRSGHIHHHDVRVAEH-----------------HV

D._rerio_Cdc20 CLSWN---DHILSSGSRSGLIHQHDVRVADH-----------------HI

D._melanogaster_fizzy SLAWN---SFLVSSGSRDGTIVHHDVRAREH-----------------KL

A._thaliana_Cdc20.1 SLAWN---NHILTTGGMDGLIINNDVRIRSP-----------------IV

A._thaliana_Cdc20.2 SLAWN---NHILTTGGMDGLIINNDVRIRSP-----------------IV

A._thaliana_Cdc20.3 SLAWN---NHILTTGGMDGKIVNNDVRIRSS-----------------IV

A._thaliana_Cdc20.4 SLAWD---NHILTTGGMDGKIVNNDVRIRSS-----------------IV

A._thaliana_Cdc20.5 SLAWN---NHILTTGGMDGKIVNNDVRIRSS-----------------IV

Micromonas_Cdc20 ALAWN---GTQLATGSRDNTVMMHDVRIREH-----------------RT

P._yoelii_Cdc20 SLCWY---YNILTTGSRDNTIINCDVRTKDS-----------------NY

P._berghei_Cdc20 ALCWY---YNILTTGSRDKTIINCDLRTKDS-----------------SY

P._chaubaudi_Cdc20 SLCWY---YNILTTGSRDNTIINCDIRTKDS-----------------NY

P._falciparum_Cdc20 TLCWN---HNTLTTGGRDNKIINSDIRSKEI-----------------YY

P._knowlesi_Cdc20 ALGWY---YDTLTTGSKDNKIVCSDIRCKDS-----------------SY

P._vivax_Cdc20 ALDWH---YNTLSTGSRDNKIVSLDIRCRES-----------------SY

S._cerevisiae_Ama1 LFEIKS-LHFSIKNWSKRQKLEDENLIGLKL------------------H

: . . : :

S._cerevisiae_Cdc20 STWAEHTGEVCGLSYKSDGLQLASGGNDNTVMIWDTRT------------

S._pombe_Slp1 GTLQGHSSEVCGLAWRSDGLQLASGGNDNVVQIWDARS------------

L._major_Cdc20 AVLRAHETEVCGLKWSPTGAMLASGGNDNQLLLWDRRSISTGSHSS-DTS

L._infantum_Cdc20 AVLRAHETEVCGLKWSPTGAMLASGGNDNQLLLWDRRSISTGSRSS-DTS

L._braziliensis_Cdc20 SVLRAHETEVCGLKWSPTGAMLASGGNDNQLLLWDRRSISTGSHSSGDTS

T._brucei_Cdc20 WTLQAHRQSVCGLRWSPDGLRLASGGNDNQLLLWDMRTLSMN--------

T._cruzi_Cdc20 WTLRCHQQSVCGLRWSPDGVRMASGGNDNQLLLWDSRTFSVR--------

S._cerevisiae_Cdh1 -TIESHTQEVCGLKWNVADNKLASGGNDNVVHVYEGTS------------

S._pombe_Srw1 -VLTAHRQEVCGLEWNSNENLLASGGNDNALMVWDKFE------------

H._sapiens_Cdh1 RRLQGHRQEVCGLKWSTDHQLLASGGNDNKLLVWNHSS------------

M._musculus_Cdh1 RRLQGHRQEVCGLKWSTDHQLLASGGNDNKLLVWNHSS------------

D._rerio_fizzy-related RRLQGHRQEVCGLKWSTDHQLLASGGNDNKLLVWNHSS------------

D._melanogaster_fizzy-related RRLAGHRQEVCGLKWSPDNQYLASGGNDNRLYVWNQHS------------

C._elegans_fzr-1 RKLTNHRQEVCGLKWSPDKQLLASGGNDNQLLVWNLRR------------

C._briggsae_Cdh1 RKMTHHRQEVCGLKWSPDKQLLASGGNDNQLLVWNLRR------------

A._thaliana_Cdh1.1 -KLKGHKSEICGLKWSSDNRELASGGNDNKLFVWNQHS------------

A._thaliana_Cdh1.2 -KLAGHKSEVCGLKWSYDNRELASGGNDNRLFVWNQHS------------

A._thaliana_Cdh1.3 -KLVGHKSEVCGLKWSHDDRELASGGNDNQLLVWNNHS------------

V._carteri_Cdc20 -KLVGHRSEVCGLKWSPDDRQLASGGNDNQLYIWSLPS------------

C._hominis_Cdc20 -RWLGHKQEVCGVKWSPNEMQLATGGNDNKLLIWSQGY------------

C._parvum_Cdc20 -RWLGHKQEVCGVKWSPNEMQLATGGNDNKLLIWSQGY------------

C._muris_Cdc20 -RWLGHKQEVCGVKWSPNEMQLATGGNDNKLLIWSQGY------------

H._sapiens_Cdc20 ATLSGHSQEVCGLRWAPDGRHLASGGNDNLVNVWP-SAPGEGGWV-----

M._musculus_Cdc20 ATLSGHSQEVCGLRWAPDGRHLASGGNDNIVNVWP-SGPGESGWA-----

D._rerio_Cdc20 FTFGGHTQEVCGLTWSPDGRYLASGGNDNMMYIWP-MTTG-SENQ-----

D._melanogaster_fizzy STLSGHTQEVCGLKWSTDFKYLASGGNDNLVNVWS-AASGGVGTAT----

A._thaliana_Cdc20.1 ETYRGHTQEVCGLKWSGSGQQLASGGNDNVVHIWD-RSVASSNSTT----

A._thaliana_Cdc20.2 ETYRGHTQEVCGLKWSGSGQQLASGGNDNVVHIWD-RSVASSNSTT----

A._thaliana_Cdc20.3 GTYLGHTEEVCGLKWSESGKKLASGGNYNVVHIWDHRSVASSKPTR----

A._thaliana_Cdc20.4 ETYLGHTEEVCGLKWSESGNKQASGGNDNVVHIWD-RSLASSKQTR----

A._thaliana_Cdc20.5 ETYLGHTEEVCGLKWSESGKKLASGGNDNVVHIWDHRSVASSNPTR----

Micromonas_Cdc20 ATLTSHSQEVCGLKWAPSGNQLASGGNDNLLHIWDQNSIGNG--------

P._yoelii_Cdc20 IKYEKHTSEVCGLQWNYNGKLLASGSNDNSIYLWDHNK------------

P._berghei_Cdc20 IKYEKHTSEVCGLQWNYNGKLLASGSNDNSIYLWDNNK------------

P._chaubaudi_Cdc20 IKYEKHTSEVCGLQWNYNGKLLASGSNDNSIYIWDNNK------------

P._falciparum_Cdc20 IELTKHKSEICGLEWNADGTYLASGSNDNSIYIWDKYT------------

P._knowlesi_Cdc20 AQLTNHTSEVCGLQWNYQTKQLASGSNDNSVYIWEWRK------------

P._vivax_Cdc20 AQLSNHSSEVCGLLWNYKTKQLASGSNDNSVCIWEERK------------

S._cerevisiae_Ama1 STYQAQAQQVCGISLNEHANLLAVGGNDNSCSLWDISD------------

: .:**: * *.* * ::

S._cerevisiae_Cdc20 -------SLPQFSKKT-HTAAVKALSWCPYSPNILASGGGQTDKHIHFWN

S._pombe_Slp1 -------SIPKFTKTN-HNAAVKAVAWCPWQSNLLATGGGTMDKQIHFWN

L._major_Cdc20 GAYRHGECQPIFFLNK-HTAAVKALSWNPTQPALLASGGGSHDKALRFWN

L._infantum_Cdc20 GVYRHGECQPIFFLNK-HTAAVKALSWNPAQPALLASGGGSHDKALRFWN

L._braziliensis_Cdc20 GIHRHGECRPIFFLNK-HTAAVKALSWNPTQPALLASGGGSHDKALRFWN

T._brucei_Cdc20 -------STPSMLLNK-HTAAVKAIAWNPVQHNLLVSGGGSDDKMLRFWN

T._cruzi_Cdc20 -------SQPVLRLNK-HTAAVKAIAWNPVQHNLLLSGGGSEDKMLRFWN

S._cerevisiae_Cdh1 -------KSPILTFDE-HKAAVKAMAWSPHKRGVLATGGGTADRRLKIWN

S._pombe_Srw1 -------EKPLYSFHN-HIAAVKAITWSPHQRGILASGGGTADRTIKLWN

H._sapiens_Cdh1 -------LSPVQQYTE-HLAAVKAIAWSPHQHGLLASGGGTADRCIRFWN

M._musculus_Cdh1 -------LSPVQQYTE-HLAAVKAIAWSPHQHGLLASGGGTADRCIRFWN

D._rerio_fizzy-related -------VLPMQQYTE-HLAAVKAIAWSPHQHGLLASGGGTADRCIRFWN

D._melanogaster_fizzy-related -------VNPVQSYTE-HMAAVKAIAWSPHHHGLLASGGGTADRCIRFWN

C._elegans_fzr-1 -------NEPIQTYTQ-HNAAVKALAWSPHHHGLLVSGGGTADRCLRFWN

C._briggsae_Cdh1 -------PDPLQTYTQ-HNAAVKALAWSPHHHGLLVSGGGTADRCLRFWN

A._thaliana_Cdh1.1 -------TQPVLRFCE-HAAAVKAIAWSPHHFGLLASGGGTADRCIRFWN

A._thaliana_Cdh1.2 -------TQPVLKYSE-HTAAVKAIAWSPHVHGLLASGGGTADRCIRFWN

A._thaliana_Cdh1.3 -------QQPILKLTE-HTAAVKAITWSPHQSSLLASGGGTADRCIRFWN

V._carteri_Cdc20 -------SSPVYKFAD-HTAAVKAIAWSPHQHSLLASGGGTADRCIRFWN

C._hominis_Cdc20 -------DTPVCQFQE-HNAAVKALSWNPHQSGLLASGGGTADRHIRIWN

C._parvum_Cdc20 -------DTPVCQFQE-HTAAVKALSWNPHQSGLLASGGGTADRHIRIWN

C._muris_Cdc20 -------ETPVCQFQE-HTAAVKALSWSPHQSGLLASGGGTADRHIRVWN

H._sapiens_Cdc20 ---------PLQTFTQ-HQGAVKAVAWCPWQSNVLATGGGTSDRHIRIWN

M._musculus_Cdc20 ---------PLQTFTQ-HQGAVKAVAWCPWQSNILATGGGTSDRHIRIWN

D._rerio_Cdc20 ---------AIHALSE-HQGAVKALAWCPWQPNIPASGGGTSDRHIRIWN

D._melanogaster_fizzy --------DPLHKFND-HQAAVRALAWCPWQPSTLASGGGTADRCIKFWN

A._thaliana_Cdc20.1 --------QWLHRLEE-HTSAVKALAWCPFQANLLATGGGGGDRTIKFWN

A._thaliana_Cdc20.2 --------QWLHRLEE-HTSAVKALAWCPFQANLLATGGGGGDRTIKFWN

A._thaliana_Cdc20.3 --------QWLHRFEE-HTAAVRALAWCPFQATLLATGGGVGDGKIKFWN

A._thaliana_Cdc20.4 --------QWLHRFEE-HTAAVRALAWCPFQASLLATGGGVGDGKIKFWN

A._thaliana_Cdc20.5 --------QWLHRFEE-HTAAVRALAWCPFQASLLATGGGVGDGKIKFWN

Micromonas_Cdc20 --------THLHRLDA-HQAAVKALAWCPFQSNLLASGGGTADRCIKFWN

P._yoelii_Cdc20 -------NNSIFHFTK-HKAAVKAISWCPHDHNLLTTGGGSADKKIYFWN

P._berghei_Cdc20 -------NNSIFHFTK-HKAAVKAISWCPHDHNLLTTGGGSTDKKIYFWN

P._chaubaudi_Cdc20 -------NDFIFHFTK-HKAAVKAISWCPHDHNLLTTGGGSADKKIYFWD

P._falciparum_Cdc20 -------NKYLFHFKK-HKAAVKAIAWCPYKNHILSSGGGSVDKKIFLWN

P._knowlesi_Cdc20 -------CVPLFQLTK-HTAAVKAMSWSPHKENLLATGGGSADKKIFLWN

P._vivax_Cdc20 -------WAPLFQFTK-HTAAVKAMSWSPHQHNLLATGGGSADKHIFFWD

S._cerevisiae_Ama1 ------LDKPIKKFVLPHKAAVKAIAFCPWSKSLLATGGGSKDRCIKFWH

* .**:*::: * :*** * : .*.

S._cerevisiae_Cdc20 SITGARVGSINTGSQVSSLHWGQSHTSTNGGMMNKEIVATGGNPENA---

S._pombe_Slp1 AATGARVNTVDAGSQVTSLIWSP-HS--------KEIMSTHGFPDNN---

L._major_Cdc20 SLTGECVHHINTGSQVCGVVWNRAGT---------ELVTAHGYTDNQ---

L._infantum_Cdc20 SLTGECVHHINTGSQVCGVVWNRVGT---------ELVTAHGYTDNQ---

L._braziliensis_Cdc20 SLTGECVHHINTGSQVCGVVWSRTGT---------ELVTAHGYTDNQ---

T._brucei_Cdc20 TSTGECISNFNAESQVCGVLWNHGGT---------ELVSSHGYSHNR---

T._cruzi_Cdc20 TSTGECINCHNAESQVCGVLWNLSGT---------ELVSSHGFSHNR---

S._cerevisiae_Cdh1 VNTSIKMSDIDSGSQICNMVWSKNTN---------ELVTSHGYSKYN---

S._pombe_Srw1 TQRGSMLHNIDTGSQVCNLLWSKQTN---------EFISTHGFMENE---

H._sapiens_Cdh1 TLTGQPLQCIDTGSQVCNLAWSKHAN---------ELVSTHGYSQNQ---

M._musculus_Cdh1 TLTGQPLQCIDTGSQVCNLAWSKHAN---------ELVSTHGYSQNQ---

D._rerio_fizzy-related TLTAQPLQCIDTGSQVCNLAWSKHTN---------ELVSTHGYSQNQ---

D._melanogaster_fizzy-related TLTGQPMQCVDTGSQVCNLAWSKHSS---------ELVSTHGYSQNQ---

C._elegans_fzr-1 TLTAQPMQCVDTGSQVCNVAWSKHSS---------ELVSTHGYSFNH---

C._briggsae_Cdh1 TLTAQPMQCVDTGSQVCNVAWSKHSS---------ELVSTHGYSYNH---

A._thaliana_Cdh1.1 TTTNTHLNCVDTNSQVCNLVWSKNVN---------ELVSTHGYSQNQ---

A._thaliana_Cdh1.2 TTTNTHLSSIDTCSQVCNLAWSKNVN---------ELVSTHGYSQNQ---

A._thaliana_Cdh1.3 TTNGNQLNSIDTGSQVCNLAWSKNVN---------EIVSTHGYSQNQ---

V._carteri_Cdc20 TATGMPLNCIDTGSQVCNISWSKNAN---------EIVSTHGYSQNQ---

C._hominis_Cdc20 TVTNSCVMAVDTGSQVCNIAWSGNVN---------ELVSTHGYSLNQ---

C._parvum_Cdc20 TVTNSCVMAVDTGSQVCNIAWSGNVN---------ELVSTHGYSLNQ---

C._muris_Cdc20 TVTNCCVMAVDTGSQVCNIAWSGNVN---------ELVSTHGYSLNQ---

H._sapiens_Cdc20 VCSGACLSAVDAHSQVCSILWSPHYK---------ELISGHGFAQNQ---

M._musculus_Cdc20 VCSGACLSAVDVHSQVCSILWSPHYK---------ELISGHGFAQNQ---

D._rerio_Cdc20 ASSGSCISSLDTCSQVSSLVFAPNYK---------ELVSGHGFAHDK---

D._melanogaster_fizzy VNNGTLMKSVDSKSQVCSLLFSRHYK---------ELISAHGFANNQ---

A._thaliana_Cdc20.1 THTGACLNSVDTGSQVCSLLWSKNER---------ELLSSHGFTQNQ---

A._thaliana_Cdc20.2 THTGACLNSVDTGSQVCSLLWSKNER---------ELLSSHGFTQNQ---

A._thaliana_Cdc20.3 THTGACLNSVETGSQVCSLLWSQRER---------ELLSSHGFTQNQ---

A._thaliana_Cdc20.4 THTGACLNSVETGSQVCSLLWSQSER---------ELLSSHGFTQNQ---

A._thaliana_Cdc20.5 THTGACLNSVETGSQVCSLLWSKSER---------ELLSSHGFTQNQ---

Micromonas_Cdc20 TNTGALLNSIDTHSQVCSLQWNKHER---------ELLSSHGYSQNQ---

P._yoelii_Cdc20 VNNGECINSINTNSQVSNILWSKNTK---------EFISTHSYTHSQ---

P._berghei_Cdc20 INNGECINSINTNSQVSNILWSKNTK---------EFISTHSYTHSQ---

P._chaubaudi_Cdc20 INNGECINSINTKSQVSNILWSKNTK---------ELISTHSYTHSQ---

P._falciparum_Cdc20 IKTGKSINEIYTKSQVSNIIWSINTS---------ELISTHSHSLNQ---

P._knowlesi_Cdc20 TSTGKCLDEVRANSQVSNIFWSKHTE---------EFVSTHSYSLGQ---

P._vivax_Cdc20 TSTGECLNELATSSQVSNLFWSKHSE---------ELVSTHSYSLGQ---

S._cerevisiae_Ama1 TSTGTLLDEIYTSGQVTSLIWSLRHK---------QIVATFGFGDTKNPV

: .*: .: : :::: .

S._cerevisiae_Cdc20 -ISVYNYETKFKVAEV-VHAHEARICCSQLSPDGTTLATVGGDENLKFYK

S._pombe_Slp1 -LSIWSYSSSGLTKQVDIPAHDTRVLYSALSPDGRILSTAASDENLKFWR

L._major_Cdc20 -LSIWRYPSLRRIANL--IGHTSRVLHLALSADGETVVSAAGDETLRFWR

L._infantum_Cdc20 -LSIWRYPSLRRIANL--IGHTSRVLHLALSADGQTVVSAAGDETLRFWR

L._braziliensis_Cdc20 -LSIWRYPSLRRIANL--IGHTSRVLHLALSADGQTVVSAAGDETLRFWR

T._brucei_Cdc20 -LTIWKYPTMRRVADL--AGHTSRVLHMCMSTDGEVVVSAAADETIRFWR

T._cruzi_Cdc20 -LTIWKYPTMRRVADL--TGHTSRVLHLCMSTDGEVVASAAGDETIRFWR

S._cerevisiae_Cdh1 -LTLWDCNSMDPIAIL--KGHSFRVLHLTLSNDGTTVVSGAGDETLRYWK

S._pombe_Srw1 -VALWNYPSVSRVGTL--KGHTDRVLYLAMSPNGENIVTGAADETLRFWK

H._sapiens_Cdh1 -ILVWKYPSLTQVAKL--TGHSYRVLYLAMSPDGEAIVTGAGDETLRFWN

M._musculus_Cdh1 -ILVWKYPSLTQVAKL--TGHSYRVLYLAMSPDGEAIVTGAGDETLRFWN

D._rerio_fizzy-related -ILVWKYPSLTQVAKL--TGHSYRVLYLAMSPDGEAIVTGAGDETLRFWN

D._melanogaster_fizzy-related -ILVWKYPSLTQVAKL--TGHSYRVLYLALSPDGEAIVTGAGDETLRFWN

C._elegans_fzr-1 -VIIWKYPSLQPVTKL--VGHQYRVLYLAMSPDGESIVTGAGDETLRFW-

C._briggsae_Cdh1 -VIIWKYPSLQPVTKL--VGHQFRVLYLAMSPDGESIVTGAGDETLRFW-

A._thaliana_Cdh1.1 -IIVWKYPTMSKLATL--TGHSYRVLYLAVSPDGQTIVTGAGDETLRFWN

A._thaliana_Cdh1.2 -IIVWKYPTMSKIATL--TGHTYRVLYLAVSPDGQTIVTGAGDETLRFWN

A._thaliana_Cdh1.3 -IMLWKYPSMSKVATL--TGHSMRVLYLATSPDGQTIVTGAGDETLRFWN

V._carteri_Cdc20 -VIIWKYPSMAKLATL--TGHTLRVLYLAVSPDGQTIVTGAGDETLRFWS

C._hominis_Cdc20 -VILWKWPSMQKIATL--TGHTYRVLYLAVSPDGQTIVTGAGDETLRFWQ

C._parvum_Cdc20 -VILWKWPSMQKIATL--TGHTYRVLYLAVSPDGQTIVTGAGDETLRFWQ

C._muris_Cdc20 -VILWKWPSMQKIVTL--TGHTYRVLYLAVSPDGQTIVTGAGDETLRFWQ

H._sapiens_Cdc20 -LVIWKYPTMAKVAEL--KGHTSRVLSLTMSPDGATVASAAADETLRLWR

M._musculus_Cdc20 -LVIWKYPTMAKVAEL--KGHTARVLGLTMSPDGATVASAAADETLRLWR

D._rerio_Cdc20 -VVIWKYPSFAKVTEH--EGHEARILNLALSPDGSTLASIAADETIRLWK

D._melanogaster_fizzy -LTIWKYPTMVKQADL--TGHTSRVLQMAMSPDGSTVISAGADETLRLWN

A._thaliana_Cdc20.1 -LTLWKYPSMVKMAEL--TGHTSRVLYMAQSPDGCTVASAAGDETLRFWN

A._thaliana_Cdc20.2 -LTLWKYPSMVKMAEL--TGHTSRVLYMAQSPDGCTVASAAGDETL----

A._thaliana_Cdc20.3 -LTLWKYPSMSKMAEL--NGHTSRVLFMAQSPNGCTVASAAGDENLRLWN

A._thaliana_Cdc20.4 -LTLWKYPSMSKMAEL--NGHTSRVLFMAQSPNGCTVASAAGDENLRLWN

A._thaliana_Cdc20.5 -LTLWKYPSMVKMAEL--NGHTSRVLFMAQSPDGCTVASAAGDETLRLWN

Micromonas_Cdc20 -LCLWKYPTMTKMAEL--TGHSARVLHMAQSPDGTTVVSAAADETLRFWK

P._yoelii_Cdc20 -IIIWNYPNLNKISAL--TDHKLRVLYAALSPDGTSLVSGSPDETIRLWN

P._berghei_Cdc20 -IIIWNYPDLNKISAL--TDHKLRVLYAALSPDGTSLVSGSPDETIRLWN

P._chaubaudi_Cdc20 -IIIWNYPDLNKISAL--TDHKLRVLYAALSPDGTSLVSGSPDETIRLWN

P._falciparum_Cdc20 -IILWNLPQLKKVTTL--RGHKSRVLYAALSPDGTSIATGSPDQTIRLWN

P._knowlesi_Cdc20 -VVLWKYPRLKKVSAL--SGHALRVLYGALSPDGESIVTGSPDETLRLWR

P._vivax_Cdc20 -VVLWKYPRLQKVSTL--SGHALRVLYGALSPDGESLVTGSPDETLRLWR

S._cerevisiae_Ama1 LITLYSYPKLSKLLEVR-SPNPLRVLSAVISPSSMAICVATNDETIRFYE

: :: : *: * .. : *:.:

S._cerevisiae_Cdc20 ------

S._pombe_Slp1 ------

L._major_Cdc20 ------

L._infantum_Cdc20 ------

L._braziliensis_Cdc20 ------

T._brucei_Cdc20 ------

T._cruzi_Cdc20 ------

S._cerevisiae_Cdh1 ------

S._pombe_Srw1 ------

H._sapiens_Cdh1 ------

M._musculus_Cdh1 ------

D._rerio_fizzy-related ------

D._melanogaster_fizzy-related ------

C._elegans_fzr-1 ------

C._briggsae_Cdh1 ------

A._thaliana_Cdh1.1 ------

A._thaliana_Cdh1.2 ------

A._thaliana_Cdh1.3 ------

V._carteri_Cdc20 ------

C._hominis_Cdc20 I-----

C._parvum_Cdc20 I-----

C._muris_Cdc20 ------

H._sapiens_Cdc20 ------

M._musculus_Cdc20 ------

D._rerio_Cdc20 ------

D._melanogaster_fizzy ------

A._thaliana_Cdc20.1 ------

A._thaliana_Cdc20.2 ------

A._thaliana_Cdc20.3 ------

A._thaliana_Cdc20.4 ------

A._thaliana_Cdc20.5 ------

Micromonas_Cdc20 ------

P._yoelii_Cdc20 ------

P._berghei_Cdc20 V-----

P._chaubaudi_Cdc20 ------

P._falciparum_Cdc20 I-----

P._knowlesi_Cdc20 ------

P._vivax_Cdc20 ------

S._cerevisiae_Ama1 LWNDKE
