# Supplementary figures and images for: A Putative Homologue of CDC20/CDH1 in the Malaria Parasite Is Essential for Male Gamete Development
Source: PLoS Pathog. 2012 Feb 23;8(2):e1002554. doi: 10.1371/journal.ppat.1002554 (PMC3285604; doi:10.1371/journal.ppat.1002554)

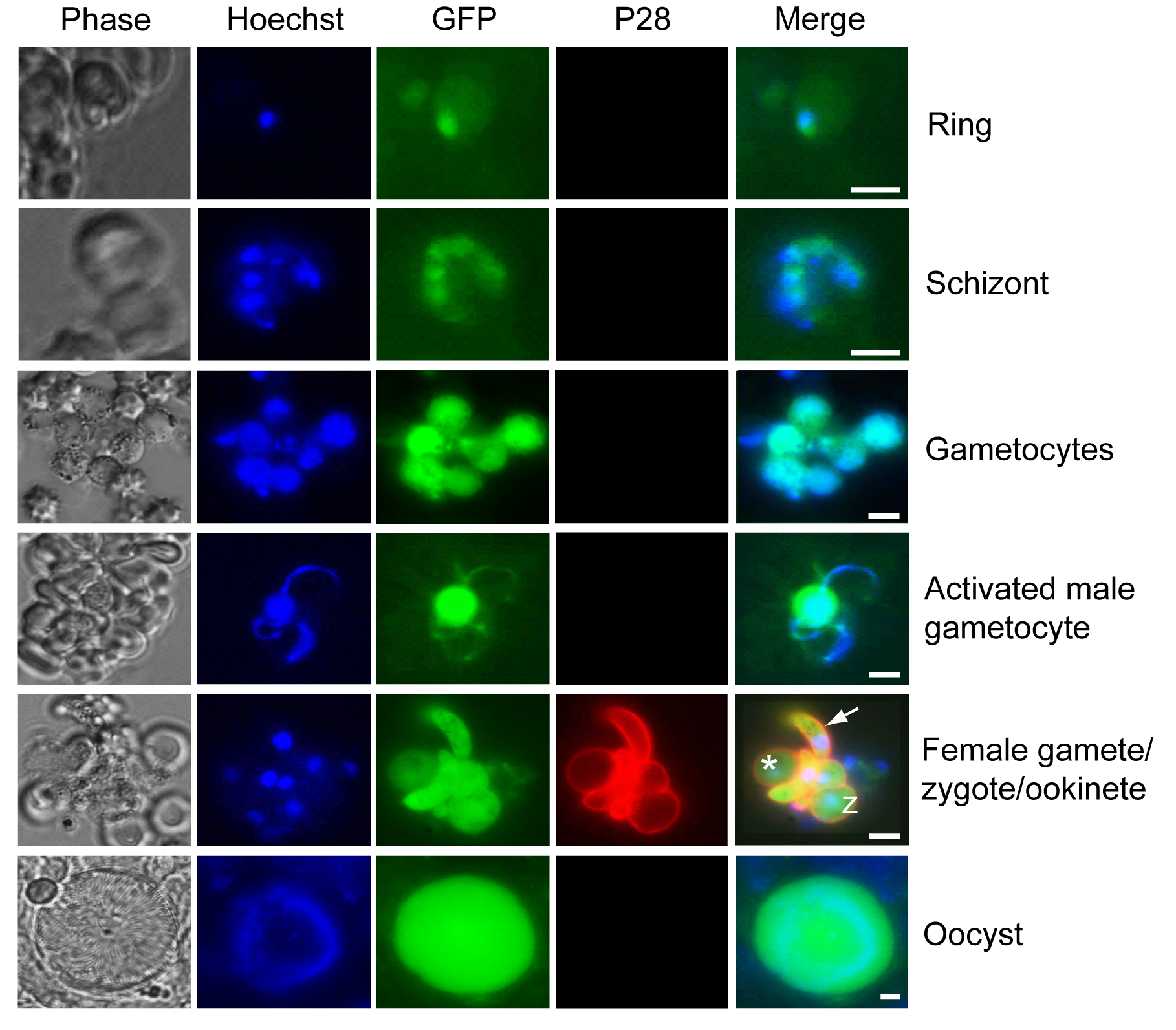

Supplement: Figure S3 — Episomal expression of PbCDC20-GFP. Episomal expression of CDC20-GFP throughout the life-cycle was shown to co-localise with Hoechst staining at all stages with addition cytoplasmic expression in ookinetes. High GFP intensity was observed at all stages. Bar = 5 µm. Female gametes (*), zygotes (z) and ookinetes (arrow) are indicated. (TIF) [file ppat.1002554.s003.tif]
