# Supplementary figures and images for: Correction: A Putative Homologue of CDC20/CDH1 in the Malaria Parasite Is Essential for Male Gamete Development
Source: PLoS Pathog. 2012 Mar 28;8(3):10.1371/annotation/ef70d427-0816-4a63-aeaf-874b734793b0. doi: 10.1371/annotation/ef70d427-0816-4a63-aeaf-874b734793b0 (PMC3315385; doi:10.1371/annotation/ef70d427-0816-4a63-aeaf-874b734793b0)

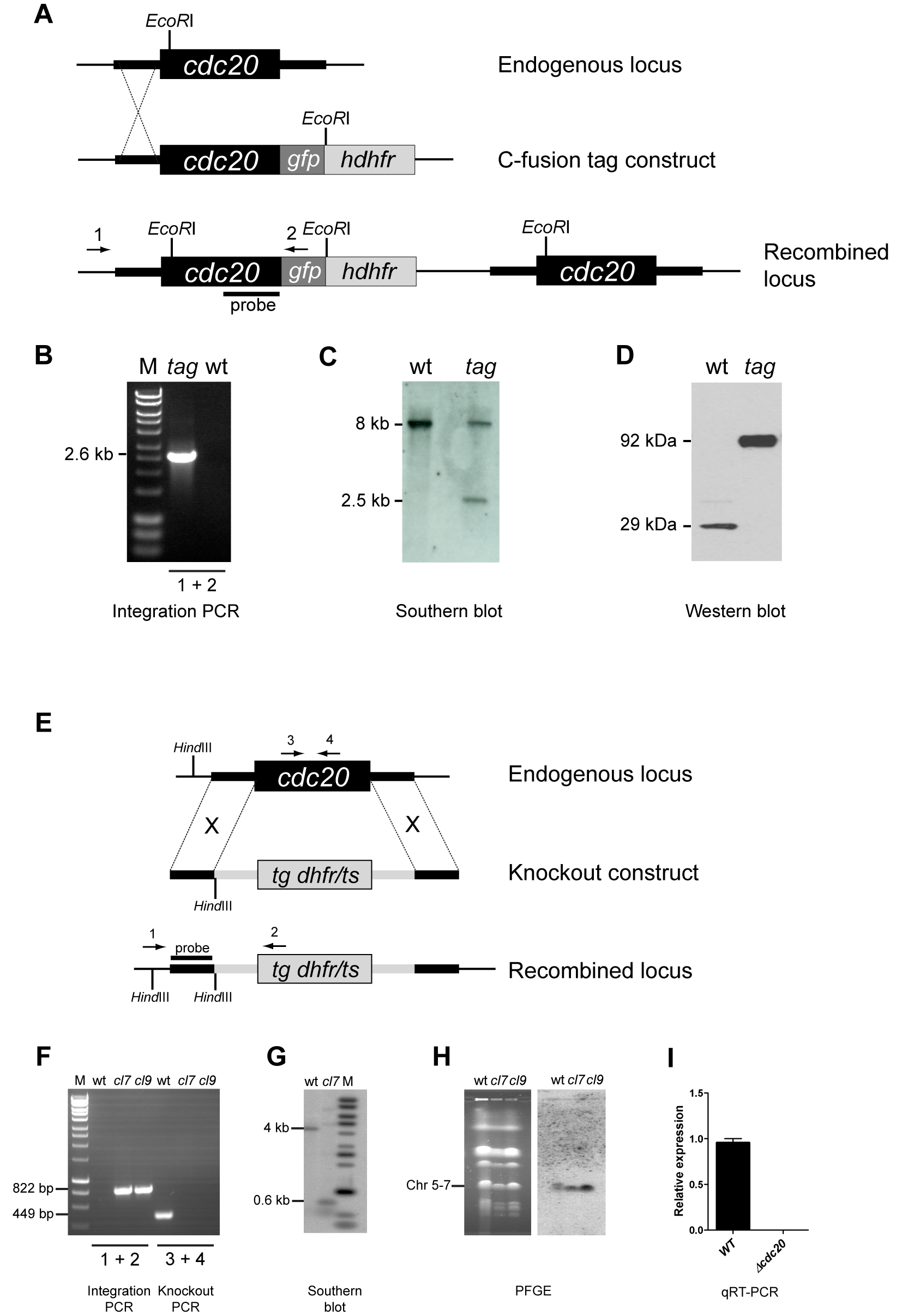

Supplement: Supplementary file 1 [file ppat.ef70d427-0816-4a63-aeaf-874b734793b0.s001.tif]
